# Missing data considerations for patient reported outcome measures in randomized controlled trials

**DOI:** 10.1186/s41687-026-01060-x

**Published:** 2026-04-11

**Authors:** Fan Wang, Shenran Deng, Jing Sun, Cunjie Lin, Shuping Jiang, Josephine M. Norquist, Gregory T. Golm, Jiaxin Xie, Yanfen Guan, William W. B. Wang, Lianzhe Xu, Hao Mei, Yang Li

**Affiliations:** 1 Biostatistics and Research Decision Sciences, MSD China, Beijing, China; 2 Center for Applied Statistics, School of Statistics, Institute of Health Data Science, Renmin University of China, Beijing, China; 3 Biostatistics and Research Decision Sciences, Patient-Centered Endpoints and Strategy, Merck & Co. Inc., Rahway, NJ USA; 4 Biostatistics and Research Decision Sciences, Late Development Statistics, Merck & Co. Inc, Rahway, NJ USA

**Keywords:** Patient-reported outcomes, Missing data, Multiple imputation, Longitudinal analysis, Clinical trials

## Abstract

**Background:**

Patient-reported outcome (PRO) measures are widely used in clinical trials to capture patients’ perspectives on their health and treatment. However, missing data remains a major challenge and can lead to biased or incomplete results. Understanding how different methods for handling missing data perform under realistic conditions is essential for decision-making.

**Methods:**

We conducted a simulation study informed by a review of 87 PRO instruments, modeling both simple and complex questionnaire structures in a hypothetical randomized trial. Under both the missing at random (MAR) and missing not at random (MNAR) assumptions, four commonly used approaches were evaluated: multiple imputation (MI) at the item level, MI at the total score level, item mean, and mixed model for repeated measurements (MMRM). To make the study more realistic, we also ran simulations using actual trial data from the COU-AA-302 study, focusing on the Functional Assessment of Cancer Therapy–Prostate (FACT-P) questionnaire.

**Results:**

The simulation results supported the use of the “Half Rule” under MAR. When the number of missing items did not exceed half of the total items, item mean imputation showed reasonable estimation with low root mean squared error, high power, and low type 1 error. MI on item scores consistently performed well, but inflated type 1 error under certain MNAR scenarios. As the missing visit rate increased, the advantage of item level imputation diminished, i.e., RMSE approached and eventually exceeded that of MI on the total score. In the trial data analysis, given the high missing visit rate in the collected FACT-P data, MI on total score level performed not worse than MI on item score level.

**Conclusions:**

No single method is optimal for all situations. Item-level imputation performs well when missing data are minimal and occur at random, but it becomes less reliable when data is missing not at random. For complex PROs or studies with many missing visits, imputing at the total score level with the “Half Rule” offers a practical alternative. Strategies should match the missingness pattern, supported by simulations and sensitivity analyses to ensure robust results.

**Supplementary Information:**

The online version contains supplementary material available at 10.1186/s41687-026-01060-x.

## Introduction

Patient-Reported Outcomes (PROs) are increasingly assessed in clinical trials as primary, secondary, or exploratory endpoints, providing the patients’ perspective of treatment and the impact of medical conditions [1]. PRO data, captured in a scientifically rigorous way, may aid clinical decision-making and clinical guideline development, support labelling claims, and influence healthcare policy [2–4]. Regulatory bodies, such as the U.S. Food and Drug Administration (FDA) [5] and European Medicines Agency (EMA) [6], actively encourage the inclusion of PRO endpoints in drug development. Health technology assessment (HTA) agencies also value PRO data as an important factor when assessing the economic, social, and ethical implications of the approval and use of a treatment [7].

In accordance with the conceptual framework of summarizing patient experiences (PFDD Guidance 3), the structure of PRO measures (PROMs) is primarily determined by the concept of interest and the context of use for the target diseases. These instruments can range from simple to complex, consisting of multiple questions and domains. This, however, introduces multiple possibilities for missingness [8–11]. In the case of multi-level PRO measures, missing data can occur at any level, including individual items, domain level summary scores, or overall total score. In addition, PRO data are commonly collected over time, resulting in a longitudinal data structure and further complicated PRO missingness.

Existing research has studied statistical methods for handling missing PRO data. Simons et al. (2014) investigated multiple imputation on item-level scores and total scores in an EQ-5D-3 L dataset from a cross-sectional study, assuming missingness at random (MAR) [12]; Rombach et al. (2016) extended the analysis scope to complex questionnaire structures by considering three PROs (OKS, SF-12, and EQ-5D-3 L) under MAR [13]. Recent studies have considered missing data handling strategies for PRO data within a longitudinal framework [11, 14–17], but they did not include item-level imputation nor consider missing not at random (MNAR). Eekhout et al. (2014) discussed the comparison of imputation methods under different missing mechanisms (MAR and MNAR) for PRO missing data, based on a single post-baseline timepoint data structure without considering item-level and total score imputations [18]. Bell et al. (2016) conducted simulation studies to investigate strategies for handling missing items in the Hospital Anxiety and Depression Scale, considering both MAR and MNAR mechanisms [19]. In their simulation settings, MNAR was assumed for domain scores rather than item-level scores. There has also been novel missing data strategy development; for example, Alacam et al. (2025) introduced factored regression models to handle item-level missing data under MAR and confirmed the importance of missing item-level scores in PRO data [20].

To the best of our knowledge, there is a lack of simulation studies that evaluate widely used, pragmatic strategies for mixed missingness patterns—including both item- and total score-level missingness—in longitudinal PRO data, while accounting for questionnaire complexity and different missing data mechanisms. This gap is important because PRO data often involve complex questionnaire structures, repeated measurements over time, and mixed patterns of missingness. To address this, our study provided a comprehensive, evidence-based evaluation of commonly used methods for handling missing-data for PRO endpoints with repeated measurements. We assessed methods performance across key practical scenarios, including: (1) repeated PRO measurements over time; (2) varying questionnaire complexity, from simple single-domain instruments to multi-domain instruments with high-level total scores; and (3) different missingness patterns (including item-level and visit-level missing data) and mechanisms (MAR or MNAR). To ensure practical relevance, we complemented the simulation experiments with analyses of data from the COU-AA-302 study using Functional Assessment of Cancer Therapy-Prostate (FACT-P) instrument. Our goal is to move beyond generic recommendations and provide context-aware guidance that supports robust PRO analysis in clinical trials.

The paper is organized as follows: Sect.  "Review of PROMs" presents a review of PROMs for the purpose of the designing hypothetical PROs in the simulation study; Sect. "Simulation"  outlines the proposed simulation study and its main findings; Sect. "Analysis on trial data" illustrates the performance of different missing data handling strategies based on the observed Functional Assessment of Cancer Therapy-Prostate (FACT-P) data from COU-AA-302; and Sect. "Discussion" summarizes the key contribution of this paper and suggestions.

## Review of PROMs

The target disease is one of the major factors impacting the content of PROMs; thus, having a comprehensive understanding of PROMs across therapeutical areas (TAs) can help design the hypothetical questionnaire in a simulation setting. Research was conducted by reviewing PRO-related statistical methods for new drug approvals by FDA from 2016 to 2022. The results provided a list of 87 PROMs in various TAs used as references to identify PROMs from different diseases. Table S1 in the Supplementary document summarizes the scoring methods by category, with each providing selected examples and Table S2 presents the review results (e.g., number of items, number of domains, item score range, total score range, and instructions on how to handle missing data) of all reviewed PROMs.

Among the 87 PROMs reviewed, the number of items per instrument varied from 1 to 50. About 17% (15 out of 87) of PROMs were single item instruments; among 72 multi-item PROMs, 33% had 2 to 10 items, 40% had 11 to 20 items, and the remaining 27% PROMs had more than 20 items. Based available information, the number of domains of a PROM was generally between 2 and 5.

For the score range of single item, both the numeric rating scale (NRS) of 0–10 and the Likert scale, which spans from 2-point to 7-point, were frequently used (58 instances of Likert scale and 14 NRS). Other options included open question and 0-100 response scales. The scoring methods were categorized into 5 distinct groups, namely: Take Mean, Take Transformed Mean, Take (Weighted/Transformed) Sum, Take Maximum, and Use Raw Score (see detailed definitions in the Supplementary document). Among these five scoring methods for computing the total score or domain score, Take (Weighted/Transformed) Sum was the most commonly utilized method (over 50%), followed by Take Mean, Take Transformed Mean, Use Raw Score, and Take Maximum.

The majority of PROMs require a minimum number of completed items to calculate a domain and/or total score. For PROMs where the total score is derived by summing all items, the requirement for missing item is typically stringent – the domain or total score is missing if any of its component is missing. For PROMs with the domain or total scores calculated by taking a mean, the commonly employed requirement is to have more than 50% of the items answered to get a non-missing score. This requirement is also known as the “Half Rule”.

The “Half Rule” is widely used in user manual of PROMs for its simplicity and feasibility in clinical trials [21, 22]. For many homogeneous domain or total scores, it may be a satisfactory way of estimating the total scores, without deleting missing data and losing statistical power. However, the validity of the “Half Rule” has been debated as well [21, 23–25]. The “Half Rule” may lead to biased estimation when item missingness is not missing completely at random (MCAR), or if the between-item correlations are small. Therefore, the validation and practical implication of “Half Rule” requires systematic investigation, to provide empirical evidence that supports context-appropriate method selection, rather than reliance on default conventions.

## Simulation

The simulation study was conducted based on a hypothetical randomized clinical trial (RCT) with hypothetical PROMs, where the change from baseline (CFB) in the PRO score is used to evaluate the treatment difference.

### Simulation settings

Patients were randomized in a 1:1 ratio into two treatment groups, Group A (treatment) and Group B (control). Assessments of PROs were scheduled at six time points: a baseline and five post-baseline visits. The treatment difference between A and B was estimated based on the CFB in the total score of the pre-defined hypothetical questionnaire, which served as the PROM, at $\:t=5$.

Based on the review results from Sect.  "Review of PROMs", two hypothetical PRO questionnaires were designed (Table 1). The first included a simple structure consisting of one domain of six items, all with identical response scales (0–10 numerical rating scale). The second included a complex structure consisting of two domains with twelve items, each with different types of response scales (5-point or 7-point Likert scales). The total score was computed by taking the mean for the simple structure and the transformed mean for the complex structure. Although Take Sum is the most commonly adopted scoring method, this method usually has little tolerance for missing items; that is, any missing item would result in a missing total score. For the hypothetical questionnaires, it was assumed that lower item, domain and total scores would indicate better health status of the subject, thus a negative treatment effect was assumed.

**Table 1:** Simulation settings: Structures of hypothetical questionnaire

Structure	Domain	No. of Items	Response Scale	Mean and Variance of Item Score at Baseline^1^	Scoring Rule of Domain Score	Domain Score Range	Scoring Rule of Total Score
Simple	Single domain	6	0–10 numerical rating scale (identical range for all items)	Item mean = 5, variance = 4, and correlation coefficient = 0.6	Mean of item score*100/range	0-100	Same as the domain score
Complex	Domain A	6	1–7 (identical range for all items)	Item mean = 4, variance = 4, and correlation coefficient = 0.6	Mean of (item score-1) *100/range	0-100	Take mean of domain scores
	Domain B	6	1–5 (identical range for all items)	Item mean = 3, variance = 2.25, and correlation coefficient = 0.6			

1. Correlations were set to obtain Cronbach's alpha > 0.7

To comprehensively examine the impact of missingness in longitudinal PRO data, thirty-nine simulation scenarios were designed, encompassing three different sample sizes, two missingness mechanisms, and combinations of missing rates and missing patterns (Table 2).

**Fig. 1:**
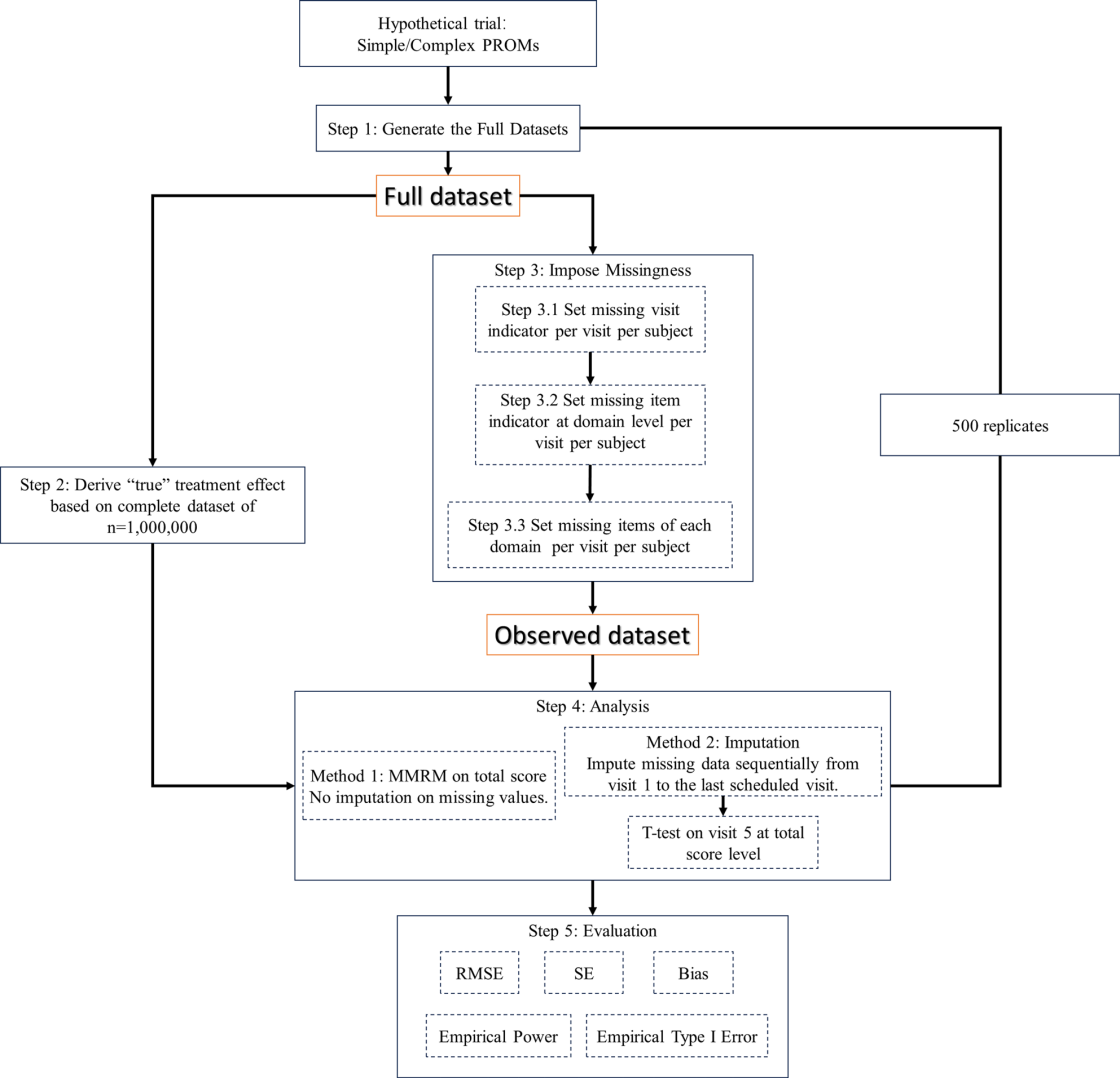
Key steps of simulation procedure: (1) generated the full datasets; (2) derive the “true” treatment effect from a large sample; (3) impose missingness; (4) analyze missing data; and (5) evaluate the analysis methods

Under each scenario, the simulation study was conducted based on the following procedure (Fig. 1): Generated a full dataset without any missingness; imposed missingness on the full data to get the observed dataset (including subjects with missing data) and complete cases dataset (excluding subjects with missing data); applied handling strategies on missing data and analysis methods to estimate the treatment effect. The missing rates and patterns were set to mimic those of the real trial data (Sect.  "Analysis on trial data"). Five-hundred replicates were conducted per scenario, as pilot simulations (Supplementary S2) indicated that results based on 500 replicates are comparable to those from 1000 replicates. The corresponding bias and type 1 error compared to the full data was close to 0 and the nominal 0.05 level, respectively. Performance was evaluated on both estimation and statistical testing. See Supplementary Figures S2.1 and  S2.2 for further details.

**Table 2:** Simulation scenarios

No. of Missing Items	Missing Rate		Simple Questionnaire		Complex Questionnaire	
	Missing Visit	Missing Item	MAR	MNAR	MAR	MNAR
Fixed (1 to 5 out of 6)	10%	30%	*N* = 200		-	
	20%	20%				
	30%	10%				
Random (Unfixed)	30%	20%	*N* = 200		*N* = 200	*N* = 100,200,400
	20%	20%	-		-	*N* = 200
	30%	10%				
	20%	10%				

Note: Definitions are stated in Sect. "Missing Data Imposition"

#### Full data generation

Subjects were randomly assigned to Group A or Group B with equal probabilities. For both simple and complex structured PRO questionnaires, the baseline item scores were generated through a multivariate normal distribution, and post-baseline item scores were generated with a non-linear model. Then domain and total scores at baseline and post-baseline visits, as well as CFB at visit 5, were calculated according to the rules specified in Table 1. The treatment effect, defined as the difference in means between two groups were − 4.39 (simple PROM) and − 4.88 (complex PROM). These numbers were obtained from estimation of treatment effect from a large complete data set (*n* = 1,000,000) in pilot simulations. For details on model specifications and calculations, refer to Supplementary Section S2.

#### Missing data imposition

Considerations on missing data included missing mechanisms, missing item rates, missing visit rates, and fixed or random number of missing items (Table 2). We considered two types of missingness: missing item and missing visit. The former one indicates that the questionnaire is partially missing, i.e., some items from the questionnaire are missing in a given visit; the latter one means that the whole questionnaire is missing in a given visit. A monotone missing pattern was assumed for missing visits, that is, if a subject has a missing visit data at a post-baseline visit, all subsequent visits were also missing. Missing items allowed intermittent missingness.

Missing rate was defined as the proportion of subjects having missing data (either missing item or missing visit) at the last visit. For missing item, two missing patterns were planned: Fixed number of missing items: the number of missing items was identical based on a pre-defined a fixed number across all visits for all subjects; Random (unfixed) number of missing items: the number of missing items was varying among subjects and visits. The former pattern was planned specially for the investigation on the impact of the number of missing items, and the latter was more consistent with real cases.

Denote $\:{I}_{it}^{visit}$ and $\:{I}_{it}^{item}$ ($\:i=1,\dots\:,n;\:t=\mathrm{0,1},\dots\:,5$) as the missing visit and missing item indicators, respectively. Here, $\:{I}_{it}^{visit}=1$ indicates that subject $\:i$ has a missing visit at time $\:t$, and $\:{I}_{it}^{item}=1$ indicates that subject $\:i$ has at least one missing item at time $\:t$. Conversely, $\:{I}_{it}^{visit}=0$ and $\:{I}_{it}^{item}=0$ means that there are no missing visit and no missing item, respectively.

Imposing missing data was conducted separately from the first post-baseline visit to the last. Missing status of each subject per visit was simulated through a Bernoulli distribution, of which the missing probability was generated by a logistic model. It was assumed that subjects with poorer health status were more likely to have missing data. Supplementary Section S2 has further details.

Missing data were generated after missing indicators were derived. For instance, if subject $\:i$ at visit $\:t$ had $\:{I}_{it}^{visit}=1$, all items scores were set to missing; if subject $\:i$ at visit $\:t$ had $\:{I}_{it}^{visit}=0$ and $\:{I}_{it}^{item}=1$, the following rule was applied: a pre-defined fixed number (or a random number) between 1 and 5 of the six items were randomly selected to be missing, with each item having an equal probability under MAR or with each item having an unequal probability (i.e., higher scores having a greater probability) under MNAR.

### Analysis methods

Mixed model for repeated measurements (MMRM), MI on item score (MI-Item), domain score (MI-Domain), or total score (MI-Total) and item mean imputation (Item-Mean) were applied to handle missing PRO data. Complete case analysis (CCA) and full-data analyses were also included as benchmarks. Item-Mean specifically addresses missing items only, whereas all other methods address both missing items and missing visits. Each strategy targets a different treatment effect under distinct missingness assumptions: MMRM estimates the marginal treatment effect on CFB using all available data, via maximum likelihood estimation without imputing missing values, and provides unbiased estimates under the assumption of MAR; MI-based methods target the same effect as MMRM, provided imputation models are correctly specified and MAR holds; and Item-Mean estimates a within-respondent average under the assumption of MCAR at the item level. In the imputation procedure, 50 imputed datasets were created in each of the 500 replicates. Imputation parameters were determined based on pilot simulations, where convergence was assessed using trace plots and potential scale reduction (PSR) statistics. The number of iterations ranges from 20 to 30 to ensure that the maximum PSR value remains below 1.10 and the 75th percentile remains below 1.05. Except for MMRM, a t-test was adopted in combination with the respective missing data handling strategies to evaluate the treatment effect. For details of method specifications, refer to Supplementary Section S2.

For scenarios with a fixed number of missing items, MI-Item, MI-Total and Item-Mean + MI-Total (i.e., Item-Mean for missing item and MI-Total for missing visit) were considered. For scenarios with a random number of missing items, a hypothetical user manual (HUM), which served as the scoring method by computing the mean of observed items if the number of non-missing items was great than or equal to three (i.e., the “Half Rule”), was employed before applying domain- or total-level imputation and MMRM. Therefore, MI-Item and HUM + MI-Total were considered for simple structured questionnaire settings, and, MI-Item, HUM + MI-Domain and HUM + MI-Total were conducted for complex structured questionnaire settings. This approach matched the common practice in clinical trials.

Methods were evaluated by root mean squared error (RMSE), standard deviance (SD), bias, empirical power and empirical type 1 error rate, at a significance level = 0.05. Details can be found in Supplementary Section S2.

We conducted all analyses in R (version 4.3.2) [26], including the R package mmrm (version 0.3.7) [27]. and multiple imputation with the R package mice (version 3.16.0) [28].

### Results

Due to the large number of simulation settings and results, we present only the main results in this subsection, with detailed results provided in Supplementary Section S2. The simulations illustrate how the performance of different methods compared and varied under different plausible missing scenarios. While results—especially under MNAR—depended on assumptions about the missingness, which cannot be fully verified, they were intended to inform rather than dictate analytic choices. The aim of these simulations was to provide evidence for context-dependent choices. Results for CCA and the full data serve only as benchmarks, to illustrate bias under naive approach (i.e., CCA) and to evaluate method performance against the truth (i.e., full data).

#### Number of missing items fixed

**Fig. 2:**
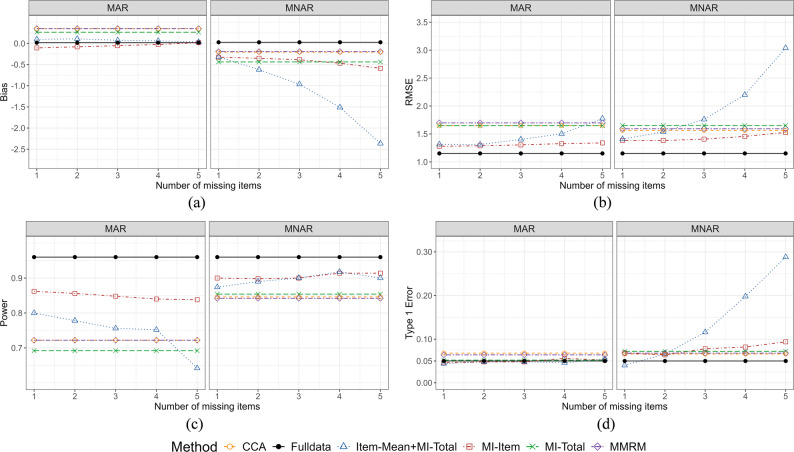
Performance metrics under simple questionnaire structure with item and visit missing rates of 0.2: bias, RMSE, power, and type 1 error rate across fixed numbers of missing items. (**a**) bias, (**b**) RMSE, (**c**) power and (**d**) type 1 error

Under the scenarios with a fixed number of missing items, simulation results supported the “Half Rule” under MAR (See Fig. 2). That is, when the number of missing items was less than or equal to half (≤ 3), the RMSE of Item-Mean + MI-Total (1.31–1.40) was relatively lower than methods which treat the summary score as missing if any item was missed, i.e., MI-Total and MMRM (RMSE 1.65 and 1.70, respectively). When the number of missing items was more than half, RMSE of Item-Mean + MI-Total steeply increased (1.50–1.78) and power similarly decreased (0.68–0.81), eventually becoming worse than MI-Total and MMRM when the number of missing items increased to five. This is because, as the number of missing items increased, the information utilized by Item-Mean decreased, causing less accurate performance.

Under MNAR, Item-Mean + MI-Total produced more biased estimates compared to other methods. This is because the missing probability of an item depends on the corresponding score under MNAR. That is, scores with higher value were more likely to be missing, causing “Item-Mean”, which takes the average of observed data, to lead to biased estimates. With the strong constrain of a fixed number of missing, Item-Mean + MI-Total performed poorly even when the number of missing items was less than three (RMSE 1.41–1.77), suggesting potential invalidity of “Half Rule” when item-level MNAR occurs.

MI-Item performed well in most scenarios (Figs. 2 and S5 – S9). It generally derived stable results with estimations close to the true value and high power under both MAR and MNAR. However, it was at risk of inflating the type 1 error (0.05–0.10), especially under MNAR and when the number of missing items was large (Figure S9). Under MNAR, the biases (MAR: -0.07–0.08 vs. MNAR − 0.57 - -0.23), RMSEs (MAR: 1.24–1.47 vs. MNAR 1.22–1.53), and type 1 error rates (MAR: 0.03–0.06 vs. MNAR 0.04–0.10) of MI-Item were higher. Besides, when the missing visit rate increased, MI-Item performed worse (Figure S5 – S9). Under MAR, RMSEs increased from 1.24 to 1.28 (missing visit rate = 0.1) to 1.45–1.47 (missing visit rate = 0.3), and powers decreased from 0.91 to 0.94 (missing visit rate = 0.1) to 0.84–0.86 (missing visit rate = 0.3). Under MNAR, RMSEs increased from 1.22 to 1.37 (missing visit rate = 0.1) to 1.44–1.52 (missing visit rate = 0.3), and power dropped from 0.96 to 0.97 (missing visit rate = 0.1) to 0.90–0.91(missing visit rate = 0.3).

By comparing the performance of different imputation and analyses methods, in most scenarios, MMRM and MI-Total did not overperform MI-Item and Item-Mean + MI-Total under MAR, especially when the number of missing items was less than half.

#### Number of missing items not fixed

Since the results from the settings of fixed numbers of missing items supported the “Half Rule” – which is the scoring algorithm adopted in many PROMs based on the review results in Sect.  "Review of PROMs" – HUM was adopted in the settings of unfixed numbers of missing items for MMRM and MI-Total. In other words, instead of treating the summary score as missing if any item was missed, the summary score was computed as the average of observed items if the number of missing times was less than half.

Similar to the settings with a fixed number of missing items in simple questionnaire structure, MI-Item performed well in most scenarios but was at risk of inflating the type 1 error under MNAR (Table 3). Under complex structured questionnaires, MI-Item showed stable and accurate estimations, with the lowest biases and RMSEs and the highest powers in most scenarios regardless of the missing mechanism (Table 3). It also demonstrated good estimation performance under different settings of missing visit rates and missing item visits, including the lowest RMSEs (MAR: 1.57 and MNAR: 1.62) and the highest power (MAR: 0.94 and MNAR: 0.93). Detailed results were presented in Table S4 and S5.

In the settings of the simple structured questionnaire, HUM + MMRM and HUM + MI-Total performed better than CCA under MAR with lower biases and RMSEs, and higher power. This revealed that, rather than removing all subjects with missing data, HUM based methods could provide added values in estimation under MAR, but may not add any value under MNAR. As the sample size increased, all methods decreased RMSE and increased power under MNAR. MI-Item consistently showed good performance, which was expected since it utilized more information. When the questionnaire was complex and the sample size was large, results from HUM + MMRM were close to those from MI-Item and were significantly better than those from summary score imputation methods (HUM + MI-Total and HUM + MI-Domain). However, the benefit of using HUM + MMRM can only be found in large samples, indicating that when the sample size is large enough (e.g., 400), especially within a complex questionnaire structure, simply adopting HUM+MMRM is not a bad choice, even MMRM by nature assumes MAR.

**Table 3:** Results under unfixed missing item numbers in simple and complex questionnaire structures with *n* = 200, missing visit rate = 0.3, missing item rate = 0.2

Questionnaire Structure	Missing Mechanism	Analysis Method	RMSE (%)^1^	Power	Type 1 Error
Simple	MAR	HUM + MMRM	1.607 (140%)	0.728	0.058
		MI-Item	**1.413 (123%)**	**0.850**	0.044
		HUM + MI-Total	1.615 (140%)	0.718	**0.042**
		CCA	1.822 (158%)	0.616	0.060
		Full Data^3^	1.152 (100%)	0.960	0.050
	MNAR	HUM + MMRM	1.950 (169%)	0.884	0.090
		MI-Item	**1.508 (131%)**	**0.891**	0.080
		HUM + MI-Total	1.762 (153%)	0.852	0.076
		CCA	1.727 (150%)	0.724	**0.050**
		Full Data	1.152 (100%)	0.960	0.050
Complex	MAR	HUM + MMRM	1.761 (132%)	0.766	0.046
		MI-Item	**1.613 (121%)**	**0.876**	0.080
		HUM + MI-Total	1.765 (132%)	0.782	**0.038**
		HUM + MI-Domain	1.620 (122%)	0.864	0.078
		CCA	1.963 (147%)	0.626	0.048
		Full Data	1.333 (100%)	0.958	0.050
	MNAR	HUM + MMRM	1.750 (131%)	0.844	**0.066**
		MI-Item	**1.660 (125%)**	0.922	0.074
		HUM + MI-Total	1.816 (136%)	0.870	0.074
		HUM + MI-Domain	1.672 (125%)	**0.924**	0.084
		CCA	1.863 (140%)	0.768	**0.066**
		Full Data	1.333 (100%)	0.958	0.050

1. The percentage in parentheses presents the ratio between the RMSE for the specific method to the RMSE for the estimations from full data;

2. The best method under a certain evaluation criterion was bolded;

3. Estimation and t-test of CFB on full data

**Fig. 3:**
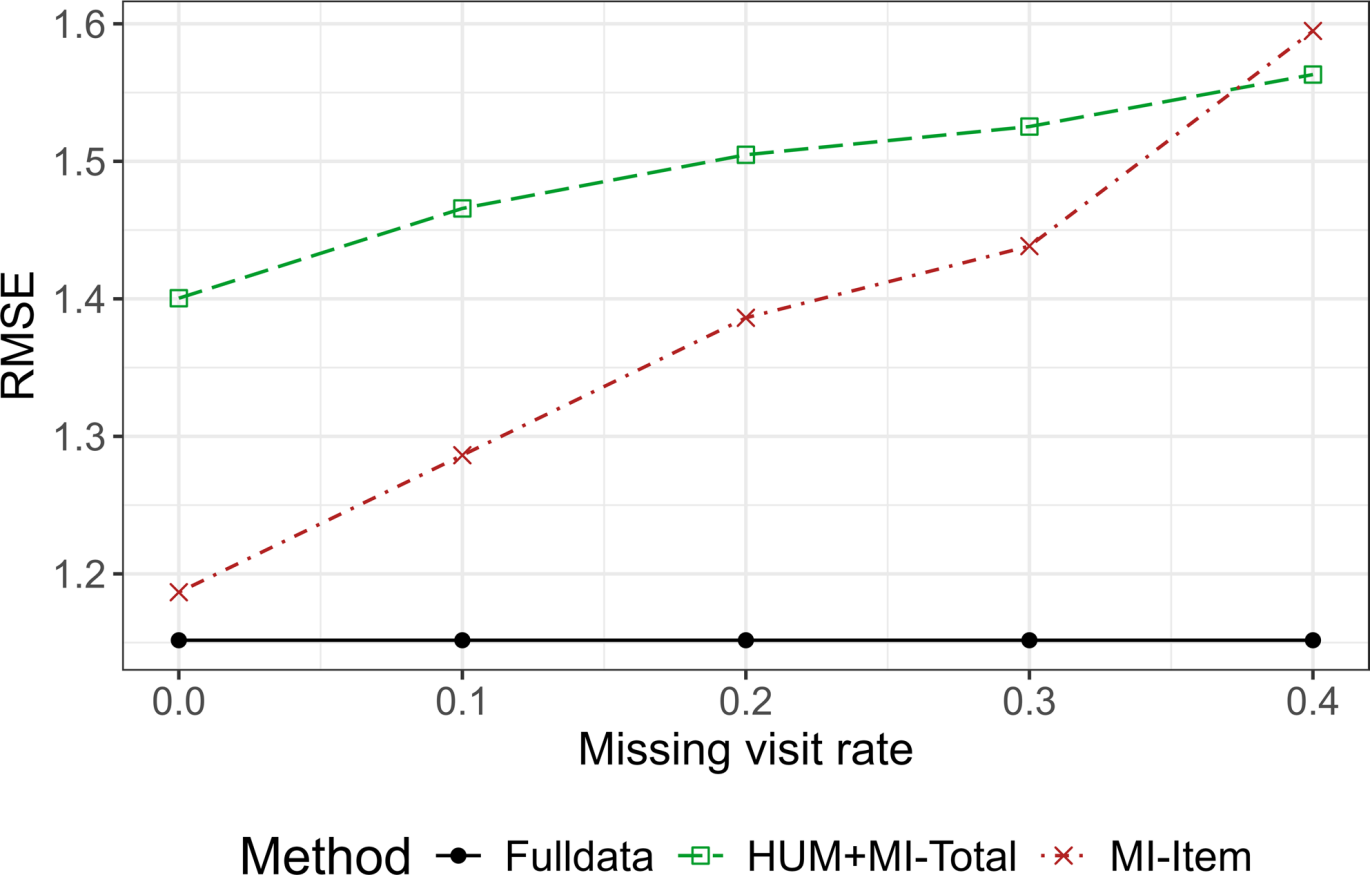
RMSEs of increasing missing visit rate with total missing rate fixed to 0.4 under MNAR and simple structured questionnaire

Given the fact that item-level and summary score-level imputations utilize different amounts of information based on the observed dataset, MI-item performed well in most scenarios. To better understand the impact of missing visit rate on item-level or total score imputation, Fig. 3 illustrates the performance (RMSE) of MI-Item and HUM + MI-Total for missing visit rate ranging from 0 to 0.4 under MNAR and in simple structured questionnaire with a fixed overall missing rate of 0.4. The RMSE of both methods increased as the missing visit rate increased (MI-Item: 1.19–1.59, HUM + MI-Total: 1.40–1.56), but MI-item had lower values when the missing visit rate was low. The two values crossed over when the missing visit rate rose to nearly 0.4.

The relationship between increasing missing visit rates and RMSE inflation was consistent across scenarios with fixed or varying numbers of missing number items (i.e., Sect.  "Number of Missing Items Fixed" and "Number of Missing Items not Fixed"). MI-Item performs better than HUM + MI-Total when visit-level missingness is low. However, its advantage decreases—and eventually reverses—as visit-level missingness increases, due to less observed data available for imputation. In summary, MI-Item is most effective when missingness is mainly sparse at the item level, but becomes less efficient under high visit dropout, even when the overall missing rate remains constant.

## Analysis on trial data

### FACT-P in COU-AA-302

Different missing data handling strategies were further evaluated by using FACT-P data collected in the COU-AA-302 study (NCT00887198) which was conducted from 2009-04-28 to 2014-03-31. The dataset was requested through the Yale University Open Data Access (YODA https://yoda.yale.edu/) platform.

The COU-AA-302 trial was a placebo-controlled, double-blind, randomized phase 3 study that enrolled 1088 asymptomatic or mildly symptomatic patients with chemotherapy-naive prostate cancer, who were stratified by Eastern Cooperative Oncology (ECOG) performance status (0 vs. 1) and randomly assigned in a 1:1 ratio to receive either abiraterone acetate (1000 mg once daily) plus prednisone (5 mg twice daily; abiraterone acetate group; *n* = 546) or placebo plus prednisone (placebo group; *n* = 542) [29]. The FACT-P questionnaire was used to assess the quality of life.

Missing baseline was not the focus of this study. For subjects with non-missing baseline (i.e., Cycle 1), the missing visit rate over time is summarized in Table 4. Cycle 7 (with missing visit rate as 26.0%) was set as the analysis time point. Missing item rate up to Cycle 7 can be found in Table 5. A majority of subjects in this dataset had ≤ 3 missing FACT-P items at each assessment.

**Table 4:** Summary of missing visits: Number (percentage) of subjects with missing visit by treatment group over time

Visit	Treatment Group, *n* (Missing Visit Rate)	Control Group, *n* (Missing Visit Rate)	Pooled, *n* (Missing Visit Rate)
Cycle 1	269 (0%)	270 (0%)	539 (0%)
Cycle 3	259 (3.7%)	252 (6.7%)	511 (5.2%)
Cycle 5	245 (8.9%)	219 (18.9%)	464 (13.9%)
**Cycle 7**	**223 (17.1%)**	**176 (34.8%)**	**399 (26.0%)**
Cycle 10	204 (24.2%)	135 (50.0%)	339 (37.1%)
Cycle 13	171 (36.4%)	102 (62.2%)	273 (49.4%)

**Table 5:** Summary of missing item: Number of subjects with missing item by treatment group, visit and the number of missing items

No. of Missing Items	Treatment Group, *n*			Control Croup, *n*		
	Cycle 3	Cycle 5	Cycle 7	Cycle 3	Cycle 5	Cycle 7
0	190	186	164	189	160	132
1	43	33	42	40	40	31
2	12	13	5	9	7	8
3	2	3	6	4	2	3
4	1	2	1	2	0	0
5	0	0	0	1	1	0
6	0	0	0	0	2	0
7	1	0	1	0	0	0
8	0	0	0	0	0	0
9	0	1	0	0	0	0
10	0	0	0	0	0	0
10+	10	7	4	7	7	2
Missing item rate	25.6%	21.9%	21.9%	23.3%	21.9%	16.3%

Two analysis datasets were defined:

- Analysis Dataset 1 (AD-1): Subjects with non-missing baseline.
- Analysis Dataset 2 (AD-2): Subjects with non-missing data from Cycle 1 to Cycle 7.

### Analysis based on AD-1

AD-1 contained 539 subjects (269 in the treatment group and 270 in the control group). Baseline characteristics of covariates and the FACT-P total score for AD-1 are summarized in Table S6. The distributions of the FACT-P total score and change scores by visit and treatment group are illustrated in Figure S11 and S12, respectively, showing similar distributions of the FACT-P total score between treatment groups.

To meet the current practice, the scoring method of FACT-P, which follows the “Half Rule”, was applied for MMRM, MI-Total and MI-Domain before conducting any missing data handling strategies on summary scores.

Referring to [30], the analysis model for both imputation and estimation incorporated with age, baseline ECOG score and baseline bone metastasis as covariates. Five methods with different missing data handling strategies were considered to estimate CFB in FACT-P total score at Cycle 7: MMRM based on the observed dataset; MI-Item + ANCOVA with MI on item-level score and ANCOVA based on imputed datasets; MI-Domain + ANCOVA with MI on domain-level score (subscale) and ANCOVA based on imputed datasets; MI-Total + ANCOVA with MI on total score and ANCOVA based on imputed datasets; CCA + ANCOVA with ANCOVA based on the observed dataset of subject with non-missing data. In order to pool analysis results from multiple imputed datasets, we fitted a univariate linear regression model (equivalently, an ANCOVA) to each imputed dataset and applied Rubin’s rules to pool the treatment effect estimates and the corresponding standard errors [31].

Table 6 summaries the analysis results. The estimated treatment effects from all methods ranged from 1.19 to 1.99, with notable differences across methods. There were moderate variations in standard errors (SE), with the MI-Item + ANCOVA method having the lowest SE. The p-values from all methods (ranged from 0.11 to 0.34) were higher than 0.05, which matches the descriptive analysis in Figure S12 because no obvious between-group difference is shown at Cycle 7 in boxplots. Although the test conclusions were consistent in this case, differences in the point estimation and SE were substantial across methods. This suggested that the choice of missing data handling strategy could potentially impact the test results.

**Table 6:** Estimation results of AD-1

	MMRM	MI-Item + ANCOVA	MI-Domain + ANCOVA	MI-Total +ANCOVA	CCA+ANCOVA
Estimation	1.59	1.76	1.19	1.99	1.47
SE	1.23	1.10	1.25	1.24	1.22
*P*-value	0.20	0.11	0.34	0.11	0.22

### Analysis based on AD-2

Since the true treatment effect was unknown for AD-1, it was impossible to evaluate the estimation accuracy. Therefore, a trial data-based simulation was conducted based on the AD-2 to further evaluate the imputation accuracy and stability of the imputation methods. While leveraging real trial data, the analysis relied on assumed missingness mechanisms and was intended to illustrate comparative performance of different methods under realistic yet hypothetical conditions. The goal was not to validate any single approach for routine use, but to offer practical insights into how these approaches may perform across plausible missingness scenarios. Figure S13 presents the data trends of the FACT-P total score by visit and treatment group for AD-2, which are similar to the those based on AD-1 (in Figure S11).

Simulation scenarios included the following: MNAR; a fixed missing item rate which was the same as the value in AD-1; two levels of missing visit rates (i.e., one was set to the actual missing visit rate in AD-1 [26%] and another was set to half of it [13%]). One hundred replicates were conducted for each scenario. MI-Item, MI-Domain, and MI-Total were employed for analysis. The summary scores of interest were the PCS subscale, TOI total score, and FACT-P total score, evaluating the analysis performance of the three methods under different complexities of the questionnaire structure.

Root mean squared imputation error (RMSIE) was utilized to evaluate the difference between the imputed value and the true value, and RMSIE% was designed for the comparison among different summary scores because these three summary scores are on different scales. A detailed definition of the evaluation criteria can be found in Supplementary Section S4.

To evaluate the impact of the “Half Rule”, RMSIE and RMSIE% were derived based on two different sets of subjects: Imputed Set #1 included subjects whose summary scores could not be calculated by the “Half Rule”; Imputed Set #2 encompassed all subjects, including those whose summary score could and could not be addressed by the “Half Rule”. Notably, in the Imputed Set #2, differences between the computed summary scores through “Half Rule” and the true values were considered as part of the imputation error. This approach allowed for an in-depth assessment of the validity of the “Half Rule” by comparing its efficacy in handling missing data across both sets.

**Fig. 4:**
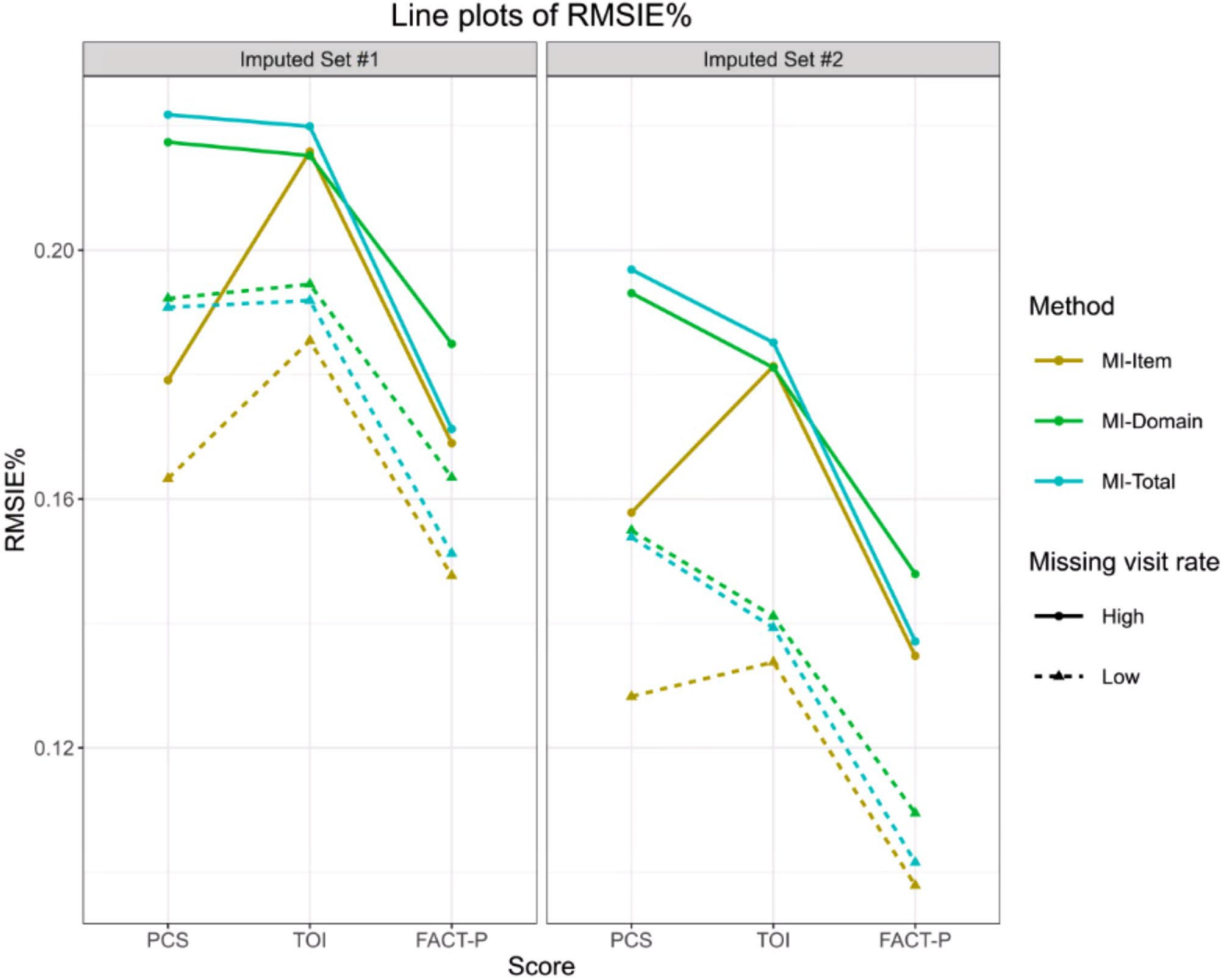
Line plots of RMSIE%

Table 7; Fig. 4 summarize the results of the three imputation methods under different scenarios. MI-Item resulted in the lowest RMSIE and RMSIE% in most scenarios, and the imputation error of MI-Domain was close to or even worse than MI-Total. Missing visit rate was a key factor significantly impacted on the performance of imputation methods; that is, the imputation error of all methods increased as the missing visit rate raised. The complexity of a summary score also had an unignorable impact on the imputation accuracy. When the structure of summary score became more complex (i.e., from prostate cancer subscale [PCS] to trial outcome index [TOI] and then to FACT-P), the relative imputation error (RMSIE%) of MI-Total and MI-Domain increased. As the structure complexity increased, the trend was not that clear for MI-Item. As the structure complexity increased, the imputation error of MI-Total and MI-Domain became close to MI-Item, i.e., RMSIE% ranged from 16.9% to 18.49% for PACT-P in Imputed Set #1 with a high missing visit rate and ranged from 17.91% to 22.18% for PCS in the same setting.

Results in AD-2 also supported the “Half Rule”. The imputation error in the Imputation Set #2 (i.e., here the impact of the “Half Rule” was considered as part of the imputation error) was significantly lower than that in the Imputation Set #1 (i.e., where the impact of the “Half Rule” was excluded), indicating the validity of the “Half Rule”. To sum up with, when the structure of the summary score is complex and the majority of subjects exhibit a small number of missing items, handling missing data with a combination of the “Half Rule” and MI-Total is sufficiently effective. This recommendation is limited to these circumstances and should not be generalized beyond this context.

**Table 7:** Analysis results of AD-2: Mean RMSIE (RMSIE%)

Subset of Subjects	Score	Missing Visit Rate	Mean of$\:{\boldsymbol{n}}_{0}$^1^	MI-Item	MI-Domain	MI-Total
Imputed Set #1	FACT-P	High	53.33	**11.64 (16.90%)**	12.75 (18.49%)	11.94 (17.13%)
		Low	27.44	**10.83 (14.77%)**	11.77 (16.35%)	11.01 (15.12%)
	TOI	High	53.17	9.13 (21.58%)	**9.07 (21.52%)**	9.37 (21.99%)
		Low	27.23	**8.27 (18.54%)**	8.48 (19.45%)	8.55 (19.19%)
	PCS	High	53.06	**4.22 (17.91%)**	4.45 (21.74%)	4.48 (22.18%)
		Low	27.19	**4.04 (16.33%)**	4.18 (19.22%)	4.15 (19.08%)
Imputed Set #2	FACT-P	High	84	**9.30 (13.48%)**	10.27 (14.79%)	9.63 (13.71%)
		Low	63	**7.20 (9.79%)**	7.99 (10.95%)	7.51 (10.16%)
	TOI	High	75.56	7.68 (18.13%)	**7.67 (18.11%)**	7.93 (18.52%)
		Low	52.83	**5.98 (13.38%)**	6.23 (14.11%)	6.28 (13.93%)
	PCS	High	69.25	**3.73 (15.78%)**	4.03 (19.31%)	4.05 (19.69%)
		Low	45.67	**3.18 (12.82%)**	3.48 (15.50%)	3.46 (18.52%)

1:  n0 denotes denotes the number of subjects with imputed values

## Discussion

### Interpretation of key findings

We investigated various strategies for handling missing data in longitudinal PRO endpoint analyses to provide insights into the underlying patterns of missingness, thereby enabling the selection of appropriate strategies for interpreting PRO endpoint data. Upon reviewing and summarizing key features of 87 PROMs, we designed a hypothetical RCT trial incorporating hypothetical PROMs of both simple and complex structures. A comprehensive simulation study was conducted based on this hypothetical trial to evaluate missing data handling strategies, including methods based on MMRM and MI on different levels. The FACT-P scores from a trial, COU-AA-302 study, were also used to further understand and compare missing data strategies in realistic scenarios. Our study highlights the importance of carefully selecting methods for handling missing data in longitudinal PRO endpoints. Through extensive experimental and trial data analysis, we offer practical guidance on choosing appropriate missing data strategies for different situations considering questionnaire structures, missing data mechanisms, missing patterns, and missing rates.

The results generally supported the “Half Rule” under MAR. When 50% or fewer items in a domain were missing, the remaining items provided meaningful interpretation, and simple approaches such as Item-Mean + MI-Total performed well. However, when more than half of the items were missing, the observed information became limited, making imputed values less reliable with inflated type 1 error and loss of power. In trial data-based simulations, missing scores imputed with Item-Mean for subjects who met the “Half Rule” were consistently more accurate than those obtained using MI-based imputation. Meanwhile, our simulation also demonstrated that when many subjects had nearly half of their items missing, performance of the “Half Rule” differed greatly in different scenarios, especially when MNAR is suspected. This can lead to substantial bias if the rule is applied without caution.

In our simulations, MI at the item score level produced more accurate, stable estimates and higher power compared to other missing data strategies. This is intuitively correct because MI-Item utilizes more information from observed data. However, this comes at the cost of higher computational burden. There were situations where the benefits of MI-Item did not justify the computational expense. For example, as the percentage of missing visits increased, or as the structure of the summary score became more complex, the performance of MI-Item approached and eventually worsened compared to MI-Total, which has much lower computational requirements. Our results also show that MI-Item may inflate type 1 error under MNAR, and MI-Total performs no worse than MI-Domain in most scenarios.

Critically, because the true missing mechanism is fundamentally unverifiable from observed data alone, it is not possible to establish a universally optimal method. Instead, our results illustrate how the performance of imputation and modeling strategies varies across missing mechanisms (e.g., MAR vs. MNAR), data structures (e.g., simple vs. complex PROMs), and missingness patterns. These findings are intended to provide evidence that supports context-aware decision-making, not one-size-fits-all rules. Furthermore, we emphasize that the results under MNAR depend on specific simulation assumptions and are therefore intended to be illustrative rather than definitive. For researchers who suspect MNAR, we recommend addressing missingness at the total-score level, which is consistent with recent publications [32, 33].

### Practical recommendations for PRO analysis

Our findings advocate for principled flexibility: method selection should align with study design, PRO data structure, missing pattern and rate, clinical context, and a realistic appraisal of potential missingness mechanisms. In practical applications, these can be approached through the following steps:

1. **Describe the Missing Pattern Over Time**: Begin by describing the missing pattern over time, including missing visit rates, missing item rates, and the number of missing items.
2. **Recommendations Based on Simulation Results and Analysis of Trial Data**: Based on simulation results, MI-Item without applying the “Half Rule” generally performed the best. The simulation results supported the “Half Rule” under MAR conditions and revealed its potential risk under MNAR. Additional sensitivity analyses under varying MNAR scenarios may be helpful to provide more reliable evidence. Here are some initial insights derived from the simulation study and the analysis of trial data, tailored to different missing data scenarios:
  1. High Item Missingness Case (e.g., missing visit proportion = 25%, missing item proportion = 75%, based on the overall missing rate as 40% in Figure S5 – S9): MI-Item is generally recommended. When most subjects have only a small number of missing items, the current practice of applying the “Half Rule” with MI-Total is acceptable. However, the simulation results revealed a potential risk of inflating type 1 error rate under MNAR when the number of missing items is nearly half. Notably, the results from the trial data fits this situation (i.e., the low missing visit rate setting in Table 7). Given the number of missing items from all subjects was small, the Half rule with MI-total provided competitive results in the trial data analysis.
  2. Balanced Missingness Case (e.g., missing visit proportion = 50%, missing item proportion = 50% based on the overall missing rate as 40% in Figure S5 – S9): The same recommendations apply as in the High Item Missingness Case. The results from the trial data fit this situation (i.e., the high missing visit rate setting in Table 7).
  3. High Visit Missingness Case (e.g., missing visit proportion = 75%, missing item proportion = 25% based on the overall missing rate as 40% in Figure S5 – S9): Both MI-Item and the current practice of applying the “Half Rule” with MI-Total are recommended.

## Additional considerations

1. It is important to note that the above summary is based on the simulation study and simulated trial data analysis, and is intended to provide some initial thoughts when conducting a trial. Team needs to evaluate their situations when designing the study. In special situations where the missing pattern is hard to discern – unlike the above-mentioned scenarios which can be clearly categorized – conducting a pilot investigation (e.g., trial data-based simulations based on preliminary data or phase 2 data, or other types of simulation considering different missing patterns and/or under different missing mechanisms) before finalizing the primary analysis can help determine an appropriate missing data handling strategy.
2. Operational strategies to control the number of missing items at the subject level in each visit are important. Preventive measures should be implemented during data collection to minimize all types of missingness.
3. Comparing to the total level imputation, item level imputation requires more computational resources. However, based on the simulation study, the computation time of all methods are acceptable.

When planning and monitoring trials with PROs, handling missing data requires a principled and context-driven approach. Statisticians should align imputation methods with the study design, PRO structure, and observed missingness patterns. Item-level multiple imputation (MI-Item) generally performs best, especially under high item missingness, while the “Half Rule” with MI-Total can be acceptable when missing items per subject are few. However, caution is warranted under MNAR conditions, as the “Half Rule” may inflate type I error when missingness approaches half of items. Sensitivity analyses under plausible MNAR scenarios are strongly recommended when MNAR is suspected. In practice, proactive monitoring of missing data patterns and operational measures to minimize missingness are critical. Early pilot simulations or interim assessments can help confirm the robustness of the chosen strategy and ensure reliable interpretation of trial results.

### Limitations and future directions

Selecting an appropriate imputation model for MI-based methods is challenging. Accuracy, stability, and rationality must all be considered when choosing an imputation model. Factors such as within- and between-domain correlations of item scores, temporal correlation of the same item score, baseline scores, and other covariates should all be taken into account. To ensure accuracy, stability, and rationality in our imputation models, we conducted pilot simulations for model selection, ultimately adopting the models that performed best in our analysis. The literature suggests that information criteria, such as the Akaike Information Criterion (AIC) or the Bayesian Information Criterion (BIC), can guide model selection [34].

There are several limitations to this study. First, the hypothetical PRO questionnaires we designed were considerably simpler than those used in practical settings, with fewer items and domains. The correlation structures we implemented were also simplistic, which may not fully capture the complexities of data within clinical trials. However, in trial data analysis using a real-world questionnaire, similar results were found. Second, the number of missing items was assumed to be either fixed or uniformly random in our simulation. In contrast, in the data from the FACT-P scores, most subjects had three or fewer missing items out of 39, and missing data patterns in longitudinal PRO datasets are likely to depend heavily on the specific concept of interest and the context of use for the target diseases. While it is difficult to simulate a sufficiently general missing data pattern, our settings allowed us to investigate the impact of the number of missing items on estimation. Additionally, the trial data analysis revealed that nearly half of the subjects had missing baseline data, which reduced the sample size for our analysis datasets. Although the issue of missing baseline PRO data was outside the scope of this study, it is an important consideration in clinical trials that warrants further investigation. Finally, our investigation did not explore other imputation strategies, such as factored regressions [20], principal component imputation [35] or passive imputation with parcel summaries [36].

These approaches could be systematically integrated into an extended version of our simulation framework, for example, by: (1) incorporating more realistic PRO structures; or (2) linking missingness mechanisms to latent clinical trajectories. Such extensions would enable more targeted methodological guidance for complex, real-world trials.

## Conclusion

This study underscores the importance of characterizing missing PRO data patterns to enhance the interpretation of longitudinal PRO endpoint results in clinical trials through appropriate statistical approaches. The findings support the validity of the “Half Rule” under with advising the caution when applying methods under MNAR conditions. Imputation utilizing item-level information generally demonstrated superior performance, though it carries a potential risk of inflating type 1 error under MNAR. These insights emphasize the need for careful methodological consideration in handling missing PRO data to ensure robust and reliable clinical trial outcomes.

## Supplementary Information

Below is the link to the electronic supplementary material.

Supplementary Material 1

## Data Availability

The datasets analyzed in Sect. "Simulation" are available from the corresponding author on reasonable request. The COU-AA-302 data analyzed in Sect. "Analysis on trial data" are obtained from the Yale University Open Data Access Project, which are openly available in YODA at Home - The YODA Project.

## Abbreviations

AD: Analysis dataset
AIC: Akaike information criterion
BIC: Bayesian information criterion
CCA: Complete case analysis
CFB: Change from baseline
ECOG: Eastern Cooperative Oncology
EMA: European Medicines Agency
FACT-P: Functional assessment of cancer therapy - prostate
FDA: Food and Drug Administration
HTA: Health technology assessment
HUM: Hypothetical user manual
Item-Mean: Item mean imputation
MAR: Missing at random
MI: Multiple imputation
MI-Domain: Multiple imputation on domain score
MI-Item: Multiple imputation on item score
MI-Total: Multiple imputation on total score
MMRM: Mixed model for repeated measurements
MNAR: Missing not at random
PCS: Prostate cancer subscale
PRO: Patient-reported outcome
PROM: Patient-reported outcome measure
RCT: Randomized clinical trial
RMSE: Root mean squared error
RMSIE: Root mean squared imputation error
SD: Standard deviance
SE: Standard error
TA: Therapeutical area
TOI: Ttrial outcome index
YODA: Yale University Open Data Access
