# Supplementary material for: Missing data considerations for patient reported outcome measures in randomized controlled trials
Source: J Patient Rep Outcomes. 2026 Apr 11;10:83. doi: 10.1186/s41687-026-01060-x (PMC13194809; doi:10.1186/s41687-026-01060-x)
Supplement: Supplementary file 1 — Supplementary Material 1 [file 41687_2026_1060_MOESM1_ESM.docx]

Supplementary Document

[S1. Summary of PROMs Review 1](#_Toc224053314)

[S2. Supportive Information of the Simulation Study 22](#_Toc224053315)

[Simulation Procedures 22](#_Toc224053316)

[Step 1: Generating Full Data 23](#_Toc224053317)

[Step 2: Derive “True” Treatment Effect 24](#_Toc224053318)

[Step 3: Setting Missing Pattern 24](#_Toc224053319)

[Step 4: Analysis Methods 25](#_Toc224053320)

[Step 5: Evaluation 26](#_Toc224053321)

[Pilot Simulation 27](#_Toc224053322)

[Simulation Results 29](#_Toc224053323)

[S3. Supportive Information of the Analysis on Trial Data 31](#_Toc224053324)

S1. Summary of PROMs Review

Scoring methods categories:

1. Take Mean: calculate the domain or summary score by simply taking the mean of answered items (usually applies to simple structured PROMs);
2. Take Transformed Mean: compute the summary score as the mean of answered items and transform it to a pre-defined range (e.g., KCCQ domain level score is computed by taking the mean of answered items and then transformed to a range of 0-100);
3. Take (Weighted/Transformed) Sum: calculate the summary score based on summation, using one of the following methods – (1) sum answered items directly (e.g., DLQI); (2) compute domain scores using weighted sums, then sum across domains for total score (e.g., FACT-P); (3) compute weighted sum and transform it to a pre-specified range (e.g., FACIT-Dyspnea short form); (4) compute the mean of answered items and multiply by the total number of items (e.g., R-ODS);
4. Take Maximum: calculate summary score by taking the maximum of answered items;
5. Use Raw Score (applies to single item PRO only).

#### **Table S1** Summary of scoring methods categories with selected examples

| **PRO Instrument** | **No. of Items** | **No. of Domains** | **Item**  **Score**  **Range** | **Scoring Rule** | **Total**  **Score Range** | **Missing Data: Minimum No. of Answered Items to Calculate a Non-missing Score/Number of All Items** | **Missing day: Minimum No. of Answered Days to Calculate a Non-missing Score/Number of All Days** |
| --- | --- | --- | --- | --- | --- | --- | --- |
| 1. **Take Mean (Simple Structure)** | | | | | | | |
| Psoriasis Symptoms and Signs Diary (PSSD) | 11 | 2 | 0-10 | Take mean of answered items, then multiply by 10. | 0-100 | 2/5 for Symptom domain  3/6 for Sign domain | 4/7  Average of the daily scores from the 7 days. |
| The Myeloproliferative Neoplasm Symptom Assessment Form total symptom score 2.0 (MPN-SAF TSS 2.0) | 10 | - | 0-10 | Take mean of answered items, then multiply by 10. | 0-100 | 6/10 | -- |
| The Brief Pain Inventory short form (BPI-SF) | 11 | 2 | 0-10 | Pain Severity Score: Take mean of answered subscale items; Pain Interference Score: Take mean of answered subscale items. | 0-10 | 2,3,4/4 for Pain Severity subscale (Requirement varies across trials, user manual recommends 4/4, see reference for details)  4/7 for Pain Interference subscale | -- |
| The Asthma Control Questionnaire-6 (ACQ-6) | 6 | - | 0-6 | Take mean of answered items | 0-6 | -- | -- |
| 1. **Take Weighted/Transformed mean (complex structure)** | | | | | | | |
| Kansas City Cardiomyopathy Questionnaire (KCCQ) | 23 | 7 | 1-5(21 items)  1-7 (2 items) | For domain scores, take mean of answered items, transform to 0-100.  For TSS, take mean of available summary scores.  For CSS and OSS, take mean of available summary scores. | 0-100 | For domain scores: 1/2, 1/3, 2/4, 3/6  At least 1 summary score should be available to calculate TSS, CSS, OSS. | -- |
| 36-Item Short Form Health Survey (SF-36) | 36 | 8 | 1-2 (7 items)  1-3(10 items)  1-5 (8 items)  1-6(11 items) | For domain scores, take mean of answered items, transform to 0-100.  For MCS, PCS and TS, take mean of available domain scores. | 0-100 | At least 50% of items should be answered to calculate domain scores.  For MCS, PCS: 3/4  For TS: 6/8 | -- |
| The EORTC Core Quality of Life Questionnaire (EORTC-QLQ-C30) | 30 | 15 | 1-4(28 items)  1-7 (2 items) | For domain scores, take mean of answered items, transform to 0-100. | 0-100 | At least 50% of items should be answered to calculate domain scores. | -- |
| Cystic Fibrosis Questionnaire-Revised (CFQ-R) | 50 | 12 | 1-4 | For domain scores, take mean of answered items, transform to 0-100. | 0-100 | -- | -- |
| 1. **Take Weighted/Transformed Sum** | | | | | | | |
| Dermatology Life Quality Index (DLQI) | 10 | 6 | 0-3 | Sum of answered items. | 0-30 | 9/10 | -- |
| Psoriasis Symptom Scale (PSS) | 8 | 2 | 0-10 | Sum of answered items. | 0-40 (symptom)  0-30 (sign) | If any items used in the domain scores are missing, the domain score is counted as missing. | -- |
| The Functional Assessment of Cancer Therapy-Prostate (FACT-P) | 39 | 5 | 0-4 | For domain scores, weighted sum: Observed sum weighted by inverse of proportion of observed items.  For total scores, sum all domain scores. | 0-156 | More than 50% of items should be answered to calculate domain scores. | -- |
| FACIT-Dyspnea short form | 10 | - | 0-3 | Raw score is calculated as the observed sum weighted by inverse of proportion of observed items.  Converted to a scale score according to developer's conversion table. | 27.7-75.9 | More than 50% of items should be answered to calculate the raw score. | -- |
| Rasch-Built Overall Disability Scale (R-ODS) | 24 | - | 0-2 | Take mean of answered items, then multiply by 24. (observed sum weighted by inverse of proportion of observed items) | 0-48 | At least 90% of items should be answered to calculate total score. | - |
| 1. **Take Maximum** | | | | | | | |
| Health Assessment Questionnaire-Disability Index (HAQ-DI) | 20 | 8 | 0-3 | For section score, take the **worst** score within the section  For total score, sum of 8 section/8 | 0-3 | 2/8 for total score. | -- |
| 1. **Use Raw Score (single item PRO)** | | | | | | | |
| Bristol Stool Form Scale (BSFS) | 1 | - | 1-7 | - | 1-7 | -- | 1/7 |
| Peak Pruritus Numerical Rating Scale (PP-NRS) | 1 | - | 0-10 | - | 0-10 | -- | 4/7  Average of the daily scores from the 7 days |

#### **Table S2** Summary of scoring methods of reviewed PRO instruments.

|  | **Drug [NCT Number]** | **Instrument** | | **Endpoint type** | | **Number of items** | | **Domains** | | **Item score range** | | **Total score range** | | **Scoring** | | **Missing item** | | |
| --- | --- | --- | --- | --- | --- | --- | --- | --- | --- | --- | --- | --- | --- | --- | --- | --- | --- | --- |
| Diseases of the Nervous System | | | | | | | | | | | | | | | | | | |
| 1 | Xadago (safinamide mesylate) [[NCT03753763](https://clinicaltrials.gov/show/NCT03753763)] | | Multiple System Atrophy Health-Related Quality of Life (MSA-QoL) | | CFB | | 40 | | - | | 0-4 | | 0-160 | | Sum of answered items. | | Not found in Prot/SAP | |
|  |  | | Unified Dystonia Rating Scale (UDRS) | | CFB | | 14 | | 2 domains  Severity (14 items)  Duration (14 items) | | 0-4  0-4 | | 0 to 112 | | The sum of the severity and duration factors | | If any of the factors is missing, then the total score is missing. | |
| 2 | Austedo (deutetrabenazine) [[NCT03571256](https://clinicaltrials.gov/show/NCT03571256)] | | Yale Global Tic Severity Scale (YGTSS) | | CFB | | 11 | | 3 domains | | 0-5 | | MTSS：0-25  VTSS：0-25  GSS：0-30 | | MTSS is the sum of the 5 items for motor tic severity;  VTSS is the sum of the 5 items for vocal tic severity;  The TTS is the sum of the MTSS and the VTSS. | | - | |
|  |  | | Child and Adolescent Gilles de la Tourette Syndrome – Quality of Life- scale (C&A-GTS-QOL) | | CFB | | 27 | | 6 subscales (cognitive, coprophenomena, psychological, physical, obsessive-compulsive, and ADL) | | Cognitive (questions 11, 12, 13, 14, 18, 20, 21, 23) (range: 0- 32)  Psychological (questions 15, 16, 17, 19, 25, 27) (range: 0 – 24)  Obsessive-compulsive (questions 7, 8, 9, 10) (range: 0 – 16)  Physical (questions 1, 3, 4) (range: 0 – 12)  Coprophenomena (questions 5, 6, 22) (range: 0 -12)  ADL (questions 2, 24, 26) (range: 0-12) | | 0-100 | | Scores for the six subscales are generated by summing items and, for ease of interpretation, transformation to a range of 0 to 100 (100x [(observed score - min possible score)/(max possible score - min possible score)]). | | If a response to 1 question is missing within the subscale, the missing response will be replaced with the average of the remaining responses within the subscale; if responses to 2 or more questions within a subscale are missing, the missing responses will not be replaced and the subscale score will be set to missing; if at least 1 subscale is missing then the total score will be set to missing | |
| 3 | Aimovig (erenumabaooe) [[NCT02483585](https://clinicaltrials.gov/show/NCT02483585)] | | The Migraine Physical Function Impact Diary (MPFID) | | Proportion of patients | | 13 | | 2 domains | | 1-5 | | 1-100 | | Sum of the responses and rescaled to 0 - 100 | | - | |
| 4 | Epidiolex (cannabidiol) [[NCT04590495](https://clinicaltrials.gov/show/NCT04590495)] | | Subject/Caregiver Global Impression of Change (S/CGIC) | | CFB | | 1 | | - | | 1-7 | | 1-7 | | - | | - | |
| 5 | Ajovy  (fremanezumab-vfrm) [[NCT02629861](https://clinicaltrials.gov/show/NCT02629861)] | | Patient global impression of change (PGIC) | | CFB | | 1 | | - | | 1-7 | | 1-7 | | - | | - | |
|  |  | | 2 items patient global impression of change (PHQ-2) | | CFB | | 2 | | - | | 0-3 | | 0-6 | | Sum | | - | |
|  |  | | Migraine disability assessment  (MIDAS) | | CFB | | 5 | | 3 domains | | masked | | masked | | Sum; | |  | |
|  |  | | Work productivity and activity impairment  (WPAI) | | CFB | | 5 | | 3 open-end question + 2 items on productivity | | 0-10 | | masked | | masked | |  | |
|  |  | | 9 items patient global impression of change (PHQ-9) | | CFB | | 9 | | / | | 0-3 | | 0-27 | | Sum | |  | |
|  |  | | Migraine-Specific quality of life (version 2.1) | | CFB | | 14 | | 3 domains | | 1-6 | | 0-100 | | - | |  | |
|  |  | | EQ-5D-5L | | CFB | | 6 | | Part 1:5 domains-mobility, self-care, usual activities, pain/discomfort, and mood  Part 2: visual analog scale | | Part 1: 1-5  Part 2: 0-100 | | 0-100 | | - | | - | |
| 6 | Emgality (galcanezumab-gnlm) [[NCT02614196](https://clinicaltrials.gov/show/NCT02614196)] | | Patient Global Impression of Improvement (PGI-I) | | Percentage of Participants | | 1 | | / | | 1-7 | | 1-7 | | - | | - | |
|  |  | | Migraine- Specific Quality of Life Questionnaire version 2.1 (MSQ v2.1) | | CFB | | 14 | | 3 domains | | 1-6 | | 0-100 | | Sum | | If any of the Role Function-Restrictive, Role Function-Preventive or Emotional Function domain is missing, then the total score will be missing | |
|  |  | | Migraine Disability Assessment Questionnaire (MIDAS) | | CFB | | 5 | | 3 domains | | Patients fill in the number of lost days | | >0 | | Sum | |  | |
| 7 | Firdapse (amifampridine phosphate) [[NCT02970162](https://clinicaltrials.gov/show/NCT02970162)] | | Quantitative Myasthenia Gravis (QMG) | | CFB | | 13 | | / | | 0-3 | | 0-39 | | Sum | |  | |
|  |  | | Subject Global Impression (SGI) | | CFB | | 1 | | / | | 1-7 | | 1-7 | | - | |  | |
| 8 | Sunosi (solriamfetol) [[NCT02348632](https://clinicaltrials.gov/show/NCT02348632)] | | Epworth Sleepiness Scale (ESS) | | CFB | | 8 | | - | | 0-3 | | 0-24 | | Sum | | - | |
|  |  | | Patient Global Impression of Change (PGIc) | | CFB | | 1 | | - | | 1-7 | | - | | - | | - | |
|  |  | | Functional Outcomes of Sleep Questionnaire (FOSQ-10) | | CFB | | 10 | | - | | Q1-2: 1-4 points  Q3-7: 0-4  Q8-9: 1-4  Q10: 0-4 | | - | | mean | | - | |
|  |  | | 36-Item Short Form Health Survey Version 2 (SF-36v2) | | CFB | | 36 | | 8 scales | | 1-2 (7 items)  1-3 (10 items)  1-5 (8 items)  1-6 (11 items) | | 0-100 | | For domain scores, take mean of answered items, transform to 0-100.  For MCS, PCS and TS, take mean of available domain scores. | | At least 50% of items should be answered to calculate domain scores.  For MCS, PCS: 3/4  For TS: 6/8 | |
|  |  | | EuroQoL (EQ-5D-5L) | | CFB | |  | |  | |  | |  | |  | |  | |
| 9 | Wakix (pitolisant) [[NCT01067222](https://classic.clinicaltrials.gov/show/NCT01067222)] | | Epworth Sleepiness Scale (ESS) | | CFB | | 8 | | - | | 0-3 | | 0-24 | | Sum | | - | |
| 10 | Nourianz (istradefylline) [[NCT02610231](https://clinicaltrials.gov/show/NCT02610231)] | | Patient Global Impression-Improvement (PGI-I ) | | Descriptive | | 1 | | - | | 1-5 | | 1-5 | | - | | - | |
| 11 | Reyvow (lasmiditan succinate) [[NCT02605174](https://clinicaltrials.gov/show/NCT02605174)] | | Migraine Disability Assessment (MIDAS) | | - | | 5 | | 3 domains: Work Household work Nonwork | | Patients fill in the number of lost days | | >0 | | Sum of all lost days, then categorize into 4 grades representing No disability to severe disability. | |  | |
| 12 | Xcopri (cenobomate) [[NCT01866111](https://clinicaltrials.gov/show/NCT01866111)] | | Quality of Life in Epilepsy Questionnaire (QOLIE-31-P) | | CFB | | 31 | | 7 parts | | 0-10;  1-6  1-5  1-3  0-100  A-G | | Weighted sum | |  | |  | |
| 13 | Dayvigo (lemborexant) [[NCT02783729](https://clinicaltrials.gov/show/NCT02783729)] | | Insomnia Severity Index (ISI) | | CFB | | 7 | | 4 domains | | 0-4 | | 0-28 | | sum | |  | |
|  |  | | Fatigue Severity Scale (FSS) | | CFB | | 9 | | - | | 1-7 | | 9-63 | | the sum of all responses to the 9 questions | |  | |
| 14 | Ubrelvy (ubrogepant) [[NCT02867709](https://clinicaltrials.gov/show/NCT02867709)] | | -  e-Diary | | - | |  | |  | |  | |  | |  | |  | |
| 15 | Vyepti (eptinezumabjjmr) [[NCT02974153](https://clinicaltrials.gov/show/NCT02974153)] | | Migraine Disability Assessment (MIDAS) | | CFB | | 5 | | 3 domains: Work Household work Nonwork | | 1-5A: Open question  5B: 0-10 | | >0 | | Sum of all lost days, then categorize into 4 grades representing No disability to severe disability. | | - | |
|  |  | | Patient Global Impression of Change (PGIC) | | - | | 1 | | / | | Seven responses are possible: Very Much Improved, Much Improved, Minimally Improved, No Change, Minimally Worse, Much Worse, Very Much Worse. | |  | |  | |  | |
|  |  | | Short Form Health Survey (SF-36 v 2.0) | | CFB | |  | |  | |  | |  | |  | |  | |
|  |  | | Health Related Quality of Life (EQ-5D-5L) | | - | |  | |  | |  | |  | |  | |  | |
|  |  | | Headache Impact Test (HIT-6) | | CFB | | 6 | | - | | Never=6, Rarely=8, Sometimes=10, Very Often=11, Always=13 | | 36-78 | | Sum of each response score | | Treated as missing if the response is missing for one or more questions | |
| 16 | Nurtec ODT (rimegepant sulfate) [[NCT03461757](https://clinicaltrials.gov/show/NCT03461757)] | | Migraine Quality of Life Questionnaire (MQoLQ)  e-Diary | | CFB | | 15 | | 7 domains | |  | |  | |  | |  | |
| 17 | Ongentys (opicapone) [[NCT01227655](https://classic.clinicaltrials.gov/show/NCT01227655)] | | Unified Parkinson's Disease Rating Scale(UPDRS ) | | - | | 42 | | 6 domains | | Domain 1-4  1-39: 0-4 point  40-42:0-1 point  Domain 5:  STAGE 0; STAGE 1; STAGE 1.5; STAGE 2 ;STAGE 2.5; STAGE 3;STAGE 4;STAGE 5  Domain 6:  100%,90%, 80%, 70%, 60%, 50%, 40%, 30%, 20%, 10%, 0% | | 0-199 | | Sum | | If one or two items in a scale are missing, they will be imputed with the mean of the non-missing items of that scale. | |
|  |  | | Parkinson's Disease Sleep Scale (PDSS) | | - | | 15 | | - | | 0-10 | | 0-150 | | Sum | | If one or two items are missing, they will be imputed with the mean of the non-missing items. If three or more items are missing, no imputation will be done and the score will be set to missing. | |
|  |  | | Non-motor Symptoms Scale (NMSS) | | - | | 30 | | 9 domains | | - | | 0-360 | | dimension score：Sum of the frequency*severity  total score：Sum of all domain scores | | If frequency or severity of a single item is missing, the domain score will not be calculated | |
| 18 | Olinvyk (oliceridine) [[NCT02820324](https://clinicaltrials.gov/show/NCT02820324)] | | Numeric Pain Rating Scale  (NPRS) | | CFB | | 1 | | / | | 0-10 | | 0-10 | | - | | - | |
| Blood and Blood-Forming Organs, Certain Immune Disorders(n=4) | | | | | | | | | | | | | | | | | | |
| 19 | Hemlibra (emicizumab) [[NCT03191799](https://clinicaltrials.gov/show/NCT03191799)] | | Hemophilia Adult Quality of Life (Haem-A-QoL) Questionnaire | | CFB | | 46 | | 10 domains | |  | | 0-100 | |  | |  | |
|  |  | | EuroQoL Five-Dimension-Five Levels Questionnaire (EQ-5D-5L) | | CFB | | 6 | | 2 domains | |  | | Part A: 0-1  Part B:0-100 | | Weighted sum | |  | |
| 20 | Ultomiris (ravulizumab-cwvz) [[NCT03920293](https://clinicaltrials.gov/show/NCT03920293)] | | Myasthenia Gravis Activities of Daily Living (MG-ADL) | | Percentage of Participants | | 8 | | - | | 0-3 | | 0-24 | | sum | |  | |
|  |  | | 15 Component Myasthenia Gravis Quality of Life  (MG-QOL15r) | | CFB | | 15 | | - | | 0-2 | | 0-30 | | sum | |  | |
|  |  | | Neurological Quality of Life (Neuro-QoL) | | CFB | | 19 | | - | | 1-5 | | 19-95 | | sum | |  | |
|  |  | | Quantitative Myasthenia Gravis  (QMG) | | CFB | | 13 | | 6 domains | | 0-3 | | 0-39 | | sum | |  | |
| 21 | Inrebic (fedratinib hydrochloride) [[NCT01437787](https://clinicaltrials.gov/show/NCT01437787)] | | the modified Myelofibrosis Symptom Assessment Form (MFSAF) v2.0 diary | | CFB | | 6 | | - | | 0-10 | | 0-60 | | Sum | |  | |
| 22 | Orladeyo (berotralstat) [[NCT03873116](https://clinicaltrials.gov/show/NCT03873116)] | | EuroQoL 5-dimensional, 5-level questionnaire (EQ-5D-5L) | | CFB | | Same as above | | - | | - | | - | | - | | - | |
|  |  | | Treatment Satisfaction Questionnaire for Medication (TSQM) | | CFB | | 14 | | 4 scales | | 1-7 | | 0-100 | | Scale scores are calculated for each scale and are transformed into scores ranging from 0 to 100 | | If more than 1 item is missing from any subscale, then the subscale will not be calculated. | |
|  |  | | Work Productivity and Activity Impairment Questionnaire (WPAI) | | CFB | | 6 | | - | | 1: 0-1 point  2-4: open question  5-6:0-10 point | | - | | Absenteeism: Percent work time missed due to health: 100*[Q2/(Q2+Q4)].  Presenteeism: Percent impairment while working due to health: 100*[Q5/10].  Work productivity loss: Percent overall work impairment due to problem: 100*{Q2/(Q2+Q4)+[(1-(Q2/(Q2+Q4)))x(Q5/10)]}. Activity impairment: Percent activity impairment due to health: 100*(Q6/10). | | - | |
| 23 | Taltz (ixekizumab) [[NCT04285229](https://clinicaltrials.gov/show/NCT04285229)] | | 36-Item Short-Form Health Survey (SF-36) | | CFB | | 36 | | - | | 3-6 response options per item | | 0-100 | | - | | - | |
|  |  | | European Quality of Life– 5 Dimensions 5–Level (EQ-5D-L) | | CFB | | Same as above | | - | | - | | - | | - | | - | |
|  |  | | Work Productivity and Activity Impairment Questionnaire– Spondylo arthritis (WPAI-SpA) | | CFB | | 6 | | Same as above | | Same as above | | Same as above | | - | | Same as above | |
| 24 | Siliq (brodalumab) [[NCT03331835](https://clinicaltrials.gov/show/NCT03331835)] | | Patient Health Questionnaire-8 (PHQ-8) | | Proportion & CFB | | 8 | | - | | 0-3 | | 0-24 | | Sum | | If more than 1 item missing, set the value of the scale to missing. | |
|  |  | | Dermatology Life Quality Index (DLQI) | | CFB | | 10 | | 6 domains | | 0-3 points (0 = not at all ⁄not relevant; 1 = a little; 2 = a lot; 3 = very much) | | 0-30 | | Sum | | - | |
|  |  | | Psoriasis symptom inventory (PSI) | | CFB | | 8 | | - | | 0-4 | | 0-32 | | Sum | | - | |
| 25 | Dupixent (dupilumab) [[NCT03738397](https://clinicaltrials.gov/show/NCT03738397)] | | Worst Pruritus Numerical Rating Scale (NRS) | | Proportion of subject | | 1 | | / | | 0-10 | | 0-10 | | - | | Missing values will not be imputed for the Worst Pruritus NRS. | |
| 26 | Tremfya (guselkumab) [[NCT02203032](https://clinicaltrials.gov/show/NCT02203032)] | | Investigator's Global Assessment (IGA) | | proportion | | 1 item | | - | | 0-4 points | | 0-4 points | | - | | - | |
|  | **Both instruments are not PRO, discounted in descriptive summary.** | | Psoriasis Area and Severity Index (PASI) | | proportion | |  | | 4 | | 0-4 | | 0-72 points | | Weighted sum | | - | |
| 27 | Jeuveau (prabotulinumtoxina-xvfs) [[NCT02334436](https://clinicaltrials.gov/show/NCT02334436)] | | Glabellar Line Scale (GLS) | | CFB | | 1 | | - | | 0-4 | | 0-4 | | - | | - | |
| 28 | Skyrizi (risankizumabrzaa) [[NCT02694523](https://clinicaltrials.gov/show/NCT02694523)] | | Dermatology Life Quality Index (DLQI) | |  | | 10 | | 6 domains | |  | | 0-30 | | Sum | | If 2 or more questions are left unanswered (missing), DLQI total score is treated as missing | |
|  |  | | Work Limitations Questionnaire  (WLQ) | |  | | 25 | | 4 domains | | 0-4 | | 0-100 | | converted mathematically to 0 (no limitations) and 100 | | - | |
| 29 | Qwo (collagenase clostridium histolyticum) [[NCT03428750](https://clinicaltrials.gov/show/NCT03428750) ] | | Patient Reported Photonumeric Cellulite Severity Scale (PR-PCSS) | |  | | 1 | | / | | 0-4 | | 0-4 | | - | | - | |
|  |  | | Patient Reported Cellulite Impact Scale (PR-CIS) | |  | | 6 | | - | | 0-10 | | 0-50 | | Sum | | imputed separately on the original 5-point ordinal scales (rating 0-4) within each treatment group with missing values. | |
| Diseases of the Digestive System | | | | | | | | | | | | | | | | | | |
| 30 | Trulance (plecanatide) [[NCT03120520](https://clinicaltrials.gov/show/NCT03120520)] | | Bristol Stool Form Scale (BSFS) | | CFB | | 1 | | - | | 1-7 | | 1-7 | | - | | If a subject has fewer than four days of data observed for a week, then that subject’s data should be considered missing for that entire week and the subject considered a non- responder | |
|  |  | | Patient Global Assessment (PGA) | | CFB | | 1 | | / | | 1-5 | | 1-5 | | - | | - | |
|  |  | | PATIENT ASSESSMENT OF CONSTIPATION (PAC-SYM) | | CFB | | 12 | | - | | 0-4 | | 0-48 | | Sum | | - | |
|  |  | | Patient Assessment of Constipation -Quality of Life (PAC-QOL) | | CFB | | 28 | | 5 domains | | 0-4 | | 0-112 | | Sum | | - | |
| 31 | Symproic (naldemedine tosylate) [[NCT04355169](https://clinicaltrials.gov/show/NCT04355169)] | | NPRS (Numerical Pain Rating Scale) | | CFB | | 1 item | | / | | 0-10 | | 0-10 | | - | | - | |
| 32 | Motegrity (prucalopride succinate) [[NCT01424228](https://clinicaltrials.gov/show/NCT01424228)] | | Patient Assessment of Constipation - Symptom (PAC-SYM) | | CFB | | 12 | | - | | 0-4 | | 0-48 | | Sum | | - | |
|  |  | | Patient Assessment of Constipation - Quality of Life (PAC-QOL) | | CFB | | 28 | | 5 domains | | 0-4 | | 0-112 | | sum | | - | |
|  |  | | Short Form-36 Health Survey (SF-36) | | CFB | | Same as above | |  | |  | |  | |  | |  | |
| 33 | Ibsrela (tenapanor hydrochloride) [[NCT02621892](https://clinicaltrials.gov/show/NCT02621892)] | | Bristol Stool Form Scale (BSFS) | | CFB | | Same as above | |  | |  | |  | |  | |  | |
|  |  | | Irritable Bowel Syndrome – Quality of Life Questionnaire (IBS-QOL) | | CFB | | 34 | | 10 domains | | 1-5 | | 0-100 | | summed and averaged for a total score and then transformed to a 0-100 scale | |  | |
| 34 | Pizensy (lactitol) [[NCT02819297](https://classic.clinicaltrials.gov/show/NCT02819297)] | | Bristol Stool Form Scale (BSFS)  e-Diary | | CFB | | Same as above | |  | |  | |  | |  | |  | |
| Diseases of the Genitourinary System | | | | | | | | | | | | | | | | | | |
| 35 | Orilissa  (elagolix sodium) [[NCT01931670](https://classic.clinicaltrials.gov/show/NCT01931670)] | | Daily Assessment of Dysmenorrhea (DYS) | | Percentage of patients | | 1 item | | / | | 0-3 | | 0-3 | | - | | - | |
|  |  | | Patient Global Impression of Change (PGIC) | | - | | 1 item | | / | | 1-7 | | 1-7 | | - | | - | |
|  |  | | Endometriosis Health Profile-30 (EHP-30) | | CFB | | 30 (core)  23 (supplementary) | | 5 domains | | 1-5 | | 0-100 | | Sum, transform into 0-100 | | - | |
| 36 | Gemtesa (vibegron) [[NCT03806127](https://clinicaltrials.gov/show/NCT03806127)] | | Global Improvement Scale (GIS) | | CFB | | 1 | | / | | 1-7 | | 1-7 | | - | | - | |
|  |  | | Irritable Bowel Syndrome - Quality of Life Measure (IBS – QoL) | | CFB | | 34 | | 8 domains | | 1-5 | | 0-100 | | Sum, transform into 0-100 | |  | |
|  |  | | Work Productivity and Activity Impairment (WPAL) | | CFB | | Same as above | |  | |  | |  | |  | |  | |
| Diseases of the Eye and Adnexa | | | | | | | | | | | | | | | | | | |
| 37 | Xiidra (lifitegrast) [[NCT03287635](https://clinicaltrials.gov/show/NCT03287635)] | | Dry Eye Comfort Questionnaire | | The improvement of patient | | 1 | | / | | 0-100 | | 0-100 | | - | | - | |
| Diseases of the Musculoskeletal System and Connective Tissue | | | | | | | | | | | | | | | | | | |
| 38 | Kevzara (sarilumab) [[NCT01146652](https://clinicaltrials.gov/show/NCT01146652)] | | Health Assessment Questionnaire-Disability Index (HAQ-DI) | | CFB | | 20 | | 8 categories:  Dressing and Grooming, Rising, Eating, Walking, Hygiene, Reach, Grip, and Activities | | 0-3 | | 0-3 | | The maximum score for all the questions in each category is considered as the score for the category | | - | |
| 39 | Olumiant (baricitinib) [[NCT01721057](https://clinicaltrials.gov/show/NCT01721057)] | | Health Assessment Questionnaire-Disability Index (HAQ-DI) | | CFB | | 20 | | 8 categories:  Dressing and Grooming, Rising, Eating, Walking, Hygiene, Reach, Grip, and Activities | | 0-3 | | 0-3 | | The maximum score for all the questions in each category is considered as the score for the category | | - | |
|  |  | | Functional Assessment of Chronic Illness Therapy Fatigue (FACIT-F) Scores | | CFB | | 13 | | - | | 0-4 | | 0-42 | | The mean of a transform score for answered questions is considered as the score of the category | |  | |
|  |  | | European Quality of Life-5 Dimensions-5 Level (EQ-5D-5L) | | CFB | | Same as above | |  | |  | |  | |  | |  | |
| 40 | Rinvoq (upadacitinib) [[NCT02706847](https://clinicaltrials.gov/show/NCT02706847)] | | Health Assessment Questionnaire Disability Index (HAQ-DI) | | Proportion | | 20 | | 8 categories:  Dressing and Grooming, Rising, Eating, Walking, Hygiene, Reach, Grip, and Activities | | 0-3 | | 0-3 | | The maximum score for all the questions in each category is considered as the score for the category | | - | |
|  |  | | Short Form-36 (SF-36) | |  | | Same as above | |  | |  | |  | |  | |  | |
| Diseases of the Respiratory System | | | | | | | | | | | | | | | | | | |
| 41 | Cinqair (reslizumab) [[NCT02501629](https://clinicaltrials.gov/show/NCT02501629)] | | Asthma Control Questionnaire (ACQ)-6 | | CFB | | 6 | | - | | 0-6 | | 0-6 | | Average | | - | |
| 42 | Fasenra (benralizumab) [[NCT02075255](https://classic.clinicaltrials.gov/show/NCT02075255)] | | The Asthma Control Questionnaire-6 (ACQ-6) | | Proportion | | 6 | | - | | 0-6 | | 0-6 | | Average | | - | |
|  |  | | Standardized Asthma Quality of Life Questionnaire (AQLQ) | | Proportion | | 32 | | 4 domains | | 7-point Likert scale (7 = not impaired at all - 1 = severely impaired). | | 1-7 | | Average | | - | |
| 43 | Xofluza  (baloxavir marboxil) [[NCT02954354](https://classic.clinicaltrials.gov/show/NCT02954354)] | | EuroQol–  5 Dimensions–5 Levels (EQ-5D-5L) | | CFB | | Same as above | |  | |  | |  | |  | |  | |
|  |  | | Work productivity (WP) questionnaire | | CFB | | 4 | | - | | Open question | | - | | - | |  | |
| 44 | Yupelri (revefenacin) [[NCT02512510](https://classic.clinicaltrials.gov/show/NCT02512510)] | | St. George's Respiratory Questionnaire (SGRQ) | | Proportion of patient | | 50 | | - | | Part I (Symptoms): several scales; Part II (Activity and Impacts): dichotomous (true/false) except last question (4-point Likert scale) | | 0 to 100 | | - | | - | |
| 45 | Xenleta  (lefamulin acetate) [[NCT02813694](https://classic.clinicaltrials.gov/show/NCT02813694)] | | The 12-Item Short Form Health Survey (SF-12) | | - | | 12 | | 2 parts | |  | | - | | mean | |  | |
| Mental, Behavioral, and Neurodevelopmental Disorders | | | | | | | | | | | | | | | | | | |
| 46 | Lucemyra (lofexidine hydrochloride) [[NCT01863186](https://clinicaltrials.gov/show/NCT01863186)] | | Opiate Withdrawal Scale of Gossop (SOWS-Gossop) | | CFB | | 10 | | - | | 0-4 | | 0-40 | | sum | | - | |
| 47 | Vyleesi  (bremelanotide acetate) [[NCT02338960](https://classic.clinicaltrials.gov/show/NCT02338960)] | | Female Sexual Function Index (FSFI) | | CFB | | 19 | | 6 domains | | 1-5  0-5 | | 1.2-6 | | Weighted sum | | - | |
|  |  | | Female Sexual Distress Scale - Desire/Arousal/Orgasm(FSDS-DAO) | | CFB | | 15 | | - | | 0-4 | | 0-60 | | Sum | | - | |
| Neoplasms | | | | | | | | | | | | | | | | | | |
| 48 | Rituxan Hycela (hyaluronidase, rituximab)* [[NCT01649856](https://classic.clinicaltrials.gov/show/NCT01649856)] | | Cancer Treatment Satisfaction Questionnaire (CTSQ) | | - | | 16 | | 3 | | 1-5 | | 0-100 | | - | | - | |
|  |  | | Rituximab Administration Satisfaction Questionnaire (RASQ) | | - | | 20 | | - | | - | | 0-100 | | - | | - | |
| 49 | Nubeqa (darolutamide) [[NCT02799602](https://clinicaltrials.gov/show/NCT02799602)] | | NCCN–FACT FPSI–17 | | TTE | | 17 | | 4 domains | | 0-4 | | 0-68 | | - | | Where there are missing items, subscale scores can be prorated if >50% of items on subscale are completed. If ≤50% of the items are answered for any domain, then the score of that domain is set to missing. The total score is set to missing if the related overall item response rate is ≤80%. | |
|  |  | | BPI–SF | | TTE | | 11 | | 2 domains:  pain intensity;  level of interference with function | | 0-10 | | 0-10 | | Pain Severity Score = mean of items 3-6 (pain at its worst, pain at its least, pain on the average, pain for right now)  Pain Interference Score = mean of items 9A-9G | | 1) for the pain severity score if one answer is missing then scoring will be set to missing, (2) for the pain interference score if four or more answers are missing out of the seven questions then the score will be set to missing | |
| Endocrine, Nutritional, and Metabolic Diseases | | | | | | | | | | | | | | | | | | |
| 50 | Xermelo  (telotristat etiprate) [[NCT02026063](https://clinicaltrials.gov/show/NCT02026063)] | | Quality of Life Questionnaire (QLQ-C30) | | CFB | | 30 | | 15 domains | | 28 questions answered:1 (not at all) to 4 (very much) and 2 questions on overall health/QOL answered:1 (poor) to 7 (excellent) | | 0-100 | | For domain scores, take mean of answered items, transform to 0-100. | | - |
|  |  | | Subjective Global Assessment | | CFB | | 1 | | / | | 0-10 | | 0-10 | | - | | - |
| 51 | Symdeko (ivacaftor, tezacaftor) [[NCT03559062](https://classic.clinicaltrials.gov/show/NCT03559062)] | | Cystic Fibrosis Questionnaire-Revised (CFQ-R) | | - | | 50 | | 12 domains | | 1-4 | | 0-100 | | Scaled score for a domain 100* (mean scores of all questions in the domain) -1)/3 | | - |
| 52 | Onpattro  (patisiran sodium) [[NCT01960348](https://clinicaltrials.gov/show/NCT01960348)] | | Norfolk Quality of Life - Diabetic Neuropathy Questionnaire (Norfolk QOL-DN) | | CFB | | 47 | | 5 domains | | - | | -4 to 136 | | sum of the listed questionnaire items | | A domain score is missing if more than 50% of the included items are missing.  If the scores for all 5 domains are non-missing, then Total QOL is the sum of scores of the 5 domains; however, if at least 1 of the domains is missing and at least 50% of the items (18 items) are non-missing, then Total QOL is calculated as 35 times the mean of the non- missing items, rounded to the nearest integer. Otherwise, Total QOL is deemed as missing. |
|  |  | | EuroQoLQualityof Life Questionnaire  EQ-5D-5L | | - | |  | | 5 dimensions | | 1-5 | | -0.109～1.0 | | Create an EQ-5D-5L profile | | Missing items are coded as “9” in creating patient profiles. The index value is deemed as missing when responses are missing for 1 or more of the  5 dimensions. If the entire instrument is missing, the EQ-5D-5L index value is considered as missing. |
| 53 | Tegsedi  (inotersen sodium) [[NCT01737398](https://clinicaltrials.gov/show/NCT01737398)] | | Modified Meuropathy Impairment Score | | CFB | |  | | - | | - | | - | | Subscore: average  Total score: sum of the subscore | | If both of the sub-component values are missing, the Averaged Sub-component score is missing. |
|  |  | | Norfolk Quality of Life-Diabetic Neuropathy (Norfolk QOL-DN) | | CFB | | 47 | | 5 domains | | - | | -4~124 | | summing the imputed domain scores | | if at least 50% of the questions for a domain are not missing or if at least one question is not missing for autonomic domain |
| 54 | Vyndaqel  (tafamidis meglumine) [[NCT01994889](https://clinicaltrials.gov/show/NCT01994889)] | | Kansas City Cardiomyopathy Questionnaire Overall Score (KCCQ) | | CFB | | 23 | | 7 domains | | - | | - | | - | | - |
| 55 | Scenesse (afamelanotide) [[NCT01605136](https://classic.clinicaltrials.gov/show/NCT01605136)] | | The Dermatology Life Quality Index (DLQI) | | - | | 10 | | - | | 4-point: 0-3 | | 0-30 | | Sum of all item scores | | - |
|  |  | | The Erthropoietic protoporphyria quality of life measure (EPP-QoL) | | - | | 15 | | - | | 4-point Likert: 0 to 3, − 3-0, or − 2-1 | | -10~35 | | Sum | | - |
| 56 | Trikafta (elexacaftor, tezacaftor, ivacaftor) [[NCT04105972](https://classic.clinicaltrials.gov/show/NCT04105972)] | | Cystic Fibrosis Questionnaire-Revised (CFQ-R) | | CFB | | 50 | | 12 | | 1-4 | | 0-100 | | Scaled score for a domain 100* (mean (scores of all questions in the domain) -1)/3 | | - |
| 57 | Imcivree (setmelanotide) [[NCT03287960](https://classic.clinicaltrials.gov/show/NCT03287960)] | | Daily Hunger Questionnaire | | CFB | | 3 | | - | | 0-10 | | - | | Average | | - |
| 58 | Tepezza (teprotumumab-trbw) [[NCT03298867](https://clinicaltrials.gov/show/NCT03298867)] | | Graves’ Ophthalmopathy Quality of Life (GO-QoL) questionnaire | | CFB | | 16 | | 2 subsets | | 1-3 | | 0-100 | | Sum, and transform into 0-100  Transformed score = [(sum of each score number of completed items) / (2 * number of completed items)] * 100. | | - |
| Other Categories | | | | | | | | | | | | | | | | | | |
| 59 | Akynzeo (netupitant, palonosetron) [[NCT03403712](https://clinicaltrials.gov/show/NCT03403712)] | | Functional Living Index-Emesis (FLIE) Questionnaire | | the proportion of patients | | 18 | | 2 domains：nausea and vomiting | | 1-7 | | Sum | | Sum to get the domain & total score  Domain score (in FLIE points) = (Domain score (in mm) × 0.06) + 9 | | For the calculation of the total FLIE score (i.e., sum of the nausea and vomiting domain scores), at least 12 out of the 18 FLIE items (i.e., ≥ 66% overall item response rate) must be non-missing and both the vomiting and nausea domains must be non-missing. | |
| 60 | Barhemsys (amisulpride) [[NCT01991860](https://classic.clinicaltrials.gov/show/NCT01991860)] | | Nausea Questionnaire | | Number of patients | | 1 | | / | | 0-10 | | - | | - | | - | |

# S2. Supportive Information of the Simulation Study

## Simulation Procedures


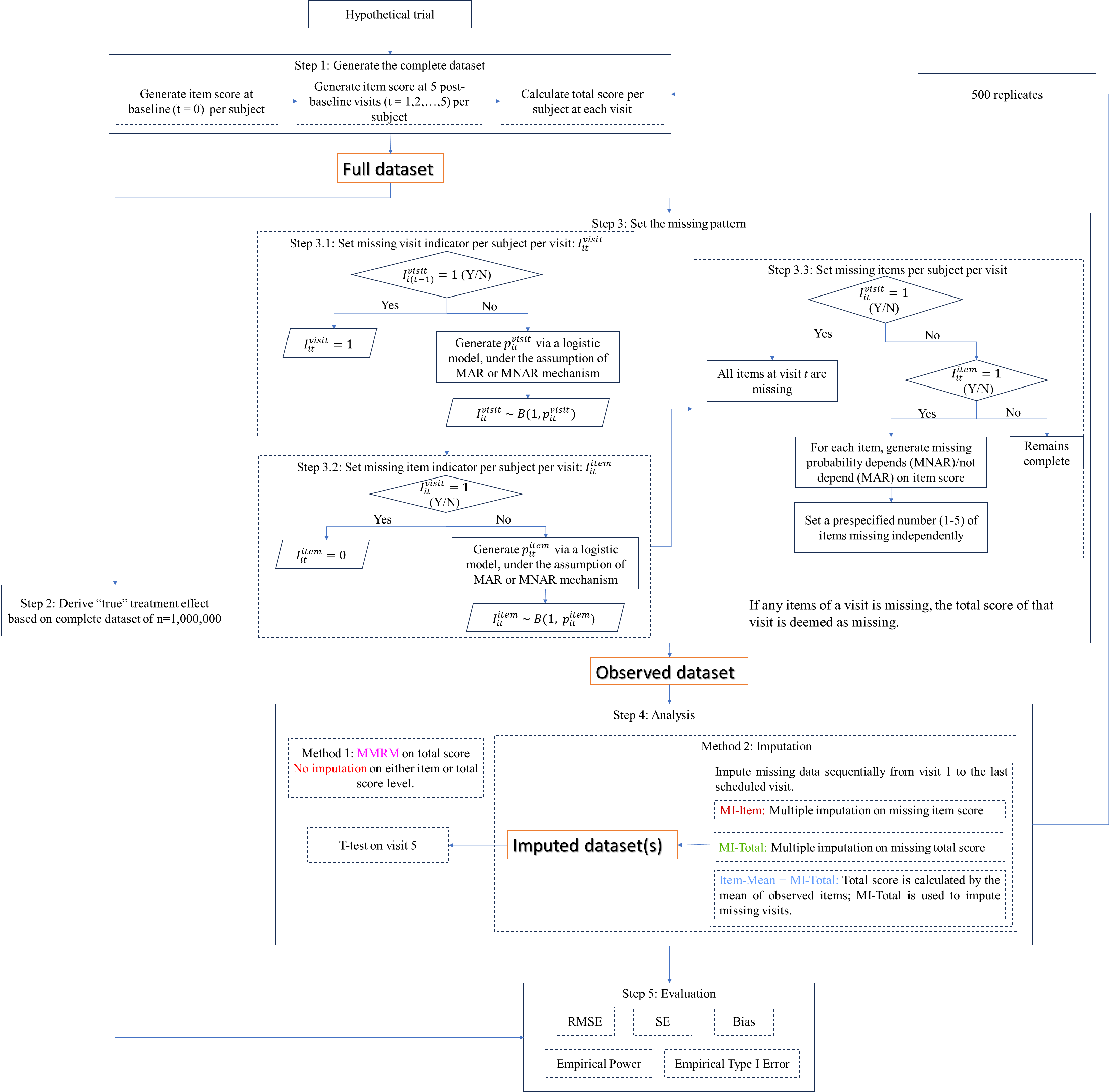


##### Figure S1 Simulation procedures of simple questionnaire structure


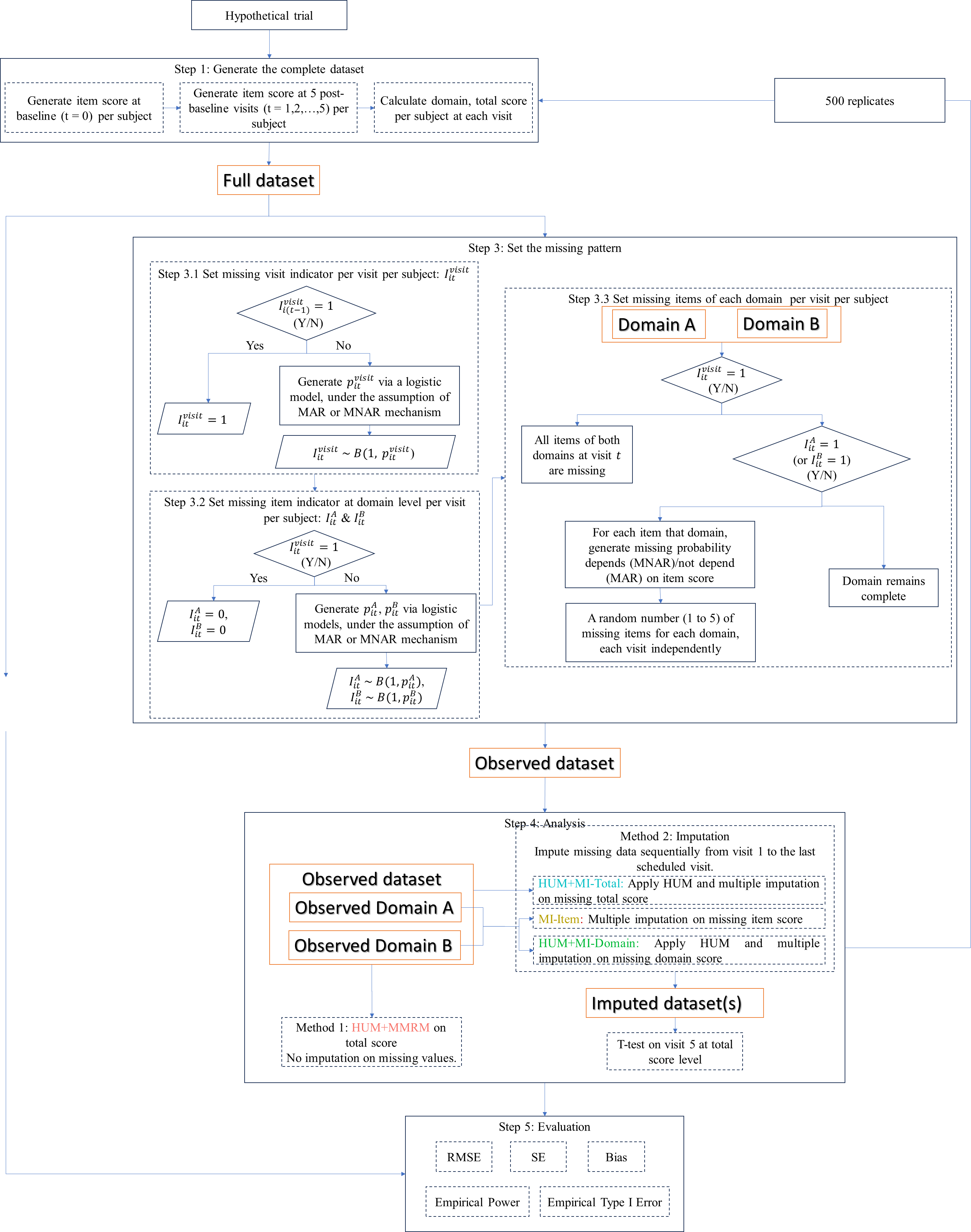


##### Figure S2 Simulation procedures of simple questionnaire structure

### Step 1: Generating Full Data

For simple structured questionnaire, let $x_{i,j,t}^{item}$ be the score, where $i=1,\ldots,n$ denotes the subject, $j=1,\ldots,6$ denotes the item and $t=0,1,\ldots,5$ denotes time point, with $t=0$ denotes the baseline. Item scores at baseline were generated from a multivariate normal distribution: $\left( x_{i,1,0}^{item},\ldots,x_{i,6,0}^{item} \right)^{T}\boldsymbol{\sim}MVN(\boldsymbol{\mu}_{\boldsymbol{1}}\mathbf{,}\boldsymbol{\Sigma}_{\mathbf{1}})$, with a mean vector $\boldsymbol{\mu}_{\boldsymbol{1}}\boldsymbol{=}{(5,\ldots, 5)}_{6\times1}^{T}$, and a variance-covariance matrix $\boldsymbol{\Sigma}_{\mathbf{1}}$, a $6\times6$ dimensional matrix with diagonal elements as 4 and off-diagonal elements as 2.4 (based on the principal of Cronbach’s alpha > 0.7), corresponding to the structures of hypothetical questionnaire in Table 1. For complex structured questionnaire, we use $x_{i,j,t}^{item}, j=1,\ldots,12$ to denote item scores as well, and $j=1,\ldots,6$ denotes items in Domain A and $j=6,\ldots,12$ denotes items in Domain B respectively. The baseline item scores were generated from a multivariate normal distribution: $\left( x_{i,1,0}^{item},\ldots,x_{i,12,0}^{item} \right)^{T}\sim MVN(\boldsymbol{\mu}_{\boldsymbol{2}}\mathbf{,}\boldsymbol{\Sigma}_{\mathbf{2}})$, where $\boldsymbol{\mu}_{\boldsymbol{2}}$ and $\boldsymbol{\Sigma}_{\mathbf{2}}$ corresponded to the settings in Table 1 as well. Post-baseline item scores were generated with a non-linear model which contains both fix and time-varying effect:

$$\begin{aligned} x_{i,j,t}^{item}=a_{0}\left( t \right)+a_{1}x_{i,j,0}^{item}+ a_{2}\left( t \right)\times treatment_{i}+\varepsilon_{i,j,t}, \end{aligned}$$

Where $a_{0}\left( t \right)$ and $a_{2}(t)$ denotes the time-dependent parameters, $a_{1}$ is the fix parameter, and $\varepsilon_{i,j,t}$ is the error term generated from a normal distribution.

Domain score of simple structured questionnaire, denoted as $x_{it}^{domain}$, were calculated as mean of items scores and transformed to 0~100. For complex structured questionnaire, let $x_{it}^{domainA}$ and $x_{it}^{domainB}$ denotes the domain score in Domain A and Domain B separately, which are calculated by taking mean of (item score – 1) and multiplied by 100/range, to transform the item means to 0~100.

The total score $x_{it}^{total}$ is the mean of domain scores, and for simple structured questionnaire, the total score is identical to the domain score. The outcome variable, change from baseline (CFB), was calculate by: ${CFB}_{i}=x_{i5}^{total}-x_{i0}^{total}$.

The mean difference of CFB between 2 groups, were deemed as the true effect of our hypothetical treatment. Since we assumed that the lower score indicates better health status, the true effect should be negative if the treatment is effective, and the effect sizes, derived from the estimation on a large complete dataset (n = 1,000,000), were -4.39 and -4.88 for simple and complex structured PROMs, respectively.

### Step 2: Derive “True” Treatment Effect

The “true” treatment effect was calculated as the mean difference with a large dataset ($n$=100,000). The “true” treatment effect for simple structured questionnaire was -4.39 and for complex structured questionnaire was -4.88.

### Step 3: Setting Missing Pattern

The missing visit indicator $I_{it}^{visit}=1$ means the $i$th subject has a missing visit at time $t$, and $I_{it}^{visit}=0$ otherwise. It was generated from binomial distribution $I_{it}^{visit}\sim B\left( 1,p_{it}^{visit} \right)$, where the missing visit probability $p_{it}^{visit}$ was generated from a logistic model:

$$\begin{aligned} p_{it}^{visit}=\frac{\exp\left( c_{0}+c_{1}treatment_{i}+c_{2}x_{i0}^{total}+c_{3}x_{it}^{total}+c_{4}t \right)}{1+\exp\left( c_{0}+c_{1}treatment_{i}+c_{2}x_{i0}^{total}+c_{3}x_{it}^{total}+c_{4}t \right)}, \end{aligned}$$

where $c_{0},c_{1},c_{2},c_{3},c_{4}$ are tuning parameters to achieve pre-specified level of missing rates and the missing mechanism. When it is unrelated to post-baseline score (i.e., $c_{3}=0$), the missing mechanism changed to MAR. It was assumed that the missing status under MNAR related to treatment, visit, baseline total score and post-baseline total score.

For those with non-missing visit (i.e. $I_{it}^{visit}\neq0$), generate missing item indicator $I_{it}^{item}\sim B(1,p_{it}^{item})$, which means subject $i$ has at least one missing item at time $t$ when $I_{it}^{item}=1$, and $I_{it}^{item}=0$ means none of items is missing. The missing item probability $p_{it}^{item}$ was derived as follows:

where $d_{0},d_{1},d_{2},d_{3},d_{4}$ are tuning parameters, and $d_{3}$ was used to determine missing mechanism (MAR or MNAR). Note that, for complex structured questionnaire, missing items were imposed separately for each domain.

For those with $I_{it}^{visit}=0$ and $I_{it}^{item}=1$, a missing probability were then generated on each item, depending (MNAR) or not depending (MAR) on its item score, and then a prespecified number (corresponding to the missing pattern that the number of missing items fixed) or a completely random number (corresponding to the missing pattern that the number of missing items unfixed) of items, were set as missing independently through a Bernoulli distribution per the missing probability of each item. Note that since we considered missing visit and missing item separately, the number of missing items was less than the total number of items in each domain.

Under the missing pattern with fixed number of missing items, we assume that, a domain score is missing as at least one item in the domain is missing, and the total score is missing as at least one item of the questionnaire is missing. This assumption helps us investigate the rationality of those methods handling missing items. Besides, if one of domain scores were missing, the total score was missing.

### Step 4: Analysis Methods

The following analysis methods were applied to evaluate and test the treatment effect:

Mixed Model for Repeated Measurements (MMRM) with treatment, visit and treatment-by-visit interaction as fixed effects, and subject as the random effect. An unstructured covariance matrix was employed to model the within-subject variance-covariance matrix. The between group difference in least square mean (LSM) with the corresponding $P$-values were derived. Note that this method assumes the missingness under MAR.

Multiple Imputation (MI) plus t-test: Multiple imputations were performed sequentially to impute missing data visit by visit. Specifically, missing data for visit 1 were imputed based on observed non-missing baseline data, missing data for visit 2 were imputed based on the imputed data from visit 1, and this process continued up to the last visit. Three MI methods were considered: imputing item-level, domain-level and total score. Fifty multiple imputations were conducted by using predictive mean matching (pmm) method to obtain imputed integer scores, as the data were collected as integers. A t-test was then applied on the imputed datasets to estimate the treatment difference, and analysis results were appropriately combined via Rubin’s rule [1] In summary, four MI-based methods were planned: multiple imputation on item score (MI-Item), domain score (MI-Domain), and total score (MI-Total), as well as item mean imputation (Item-Mean + MI-Total).

For complex structured questionnaire, information for one domain was imputed independently from others. A total of 50 imputed datasets were generated per imputation round and the number of iterations was set to 20.

1. Multiple Imputation on item score (MI-Item): For each domain, a linear regression model included all item scores at baseline and at the previous visit, other item scores at the current visit, and treatment group. For a given timepoint $t$, the imputation model for $j$th item of subject $i$ was defined as:

$$x_{i,j,t}^{item}\sim\boldsymbol{x}_{i0}^{item}+\boldsymbol{x}_{i\left( t-1 \right)}^{item}+\boldsymbol{x}_{i,\left( -j \right),t}^{item}+treatment_{i},$$

where $\boldsymbol{x}_{i0}^{item}$ denotes all of items at baseline, $\boldsymbol{x}_{i(t-1)}^{item}$ denotes all of items at the previous timepoint ($t-1)$, and $\boldsymbol{x}_{i,\left( -j \right),t}^{item}$ denoted items other than the target item at visit $t$.

If any items from $\boldsymbol{x}_{(-j)t}^{item}$ was missing, a randomly selected observed value from subjects with non-missing data for the same item in the same visit was used as the initial value, starting from the first missing item to the last, $and$ this process was updated iteratively. The same rule was applied to the situation when all items were missing (i.e., a missing visit) in a visit.

1. Multiple imputation on domain score (MI-Domain): For each domain, a linear regression model included domain score at baseline and at the previous visit, and treatment group. The imputation model was defined as:

$$x_{it}^{domain}\sim x_{i0}^{domain}+x_{i,(t-1)}^{domain}+treatment_{i}.$$

2. Multiple imputation on total score (MI-Total): The model included total score at baseline and at the previous visit, and treatment group. The imputation model can be written as:

$$x_{it}^{total}\sim x_{i0}^{total}+x_{i,(t-1)}^{total}+treatment_{i}.$$

3. Item mean imputation (Item-Mean + MI-Total): Item-Mean was applied when at least one item was missing at domain level by taking the average of observed items, regardless of the number of missing items (i.e., no need to apply the “Half Rule”), and MI-Total was employed for missing visits. This method can further investigate the impact of the number of missing items, especially when the number exceeds half of the total items within the domain.

In addition to above methods, t test will also be applied to CCA and the full dataset. All of tests were two-sided with significant level set to 0.05.

### Step 5: Evaluation

Denote $\hat{eff_{s}}$ as the estimated effect of the $s$th simulation dataset, $eff$ as the true effect derived from a large dataset (n=100,000), and $m$ as the total number of simulation datasets.

RMSE evaluated the average error between the estimated effect and the true effect:

$$\begin{aligned} RMSE=\sqrt{\frac{1}{m}\sum_{s=1}^{m} (\hat{eff_{s}}-eff)^{2}} \end{aligned}$$

SD evaluated the degree of speared out of estimates:

$$\begin{aligned} SD=\sqrt{\frac{1}{m-1}\sum_{s=1}^{m} (\hat{eff_{s}}-\overline{eff_{s}})^{2}} \end{aligned}$$

where,

$$\begin{aligned} \overline{eff_{s}}=\frac{1}{m}\sum_{s=1}^{m} \hat{eff_{s}}. \end{aligned}$$

Bias evaluated the non-random error between the estimated effect and the true effect:

$$\begin{aligned} Bias=\overline{eff_{s}}-eff. \end{aligned}$$

Empirical Power was the proportion of significant (two-sided $\alpha=0.05$) estimated effects over 500 simulated datasets. The empirical type 1 error was not applicable under the scenario with a non-zero true treatment effect. To evaluate empirical type 1 error, an additional simulation with $a_{2}(t)$ = 0 was conducted, and the proportion of significant estimated effect was the empirical type 1 error.

## Pilot Simulation

Under simple structured PROM structure, unfixed number of missing items, n =200, missing visit rate = 0.3, missing item rate = 0.2, MNAR, pilot simulations were conducted for the choice of simulation parameters (e.g., number of replicates, number of iterations and number of imputed datasets in multiple imputation). Selective results were presented to support our choices.

**Figure S3** shows the potential scale reduction (PSR) statistics [2, 3] for all 50 chains at the final observation point (*t* = 5) and the last iteration (30) in 500 replicates (simple questionnaire, MNAR, missing visit = 30%, missing item = 20%, and $n$ = 200). For the MI-Item model, the PSR for each individual item (X1–X6) ranged from 1.00 to 1.09. For the MI-Total model, the PSR for total score ranged from 0.99 to 1.02. These values indicate good convergence under the current imputation settings.


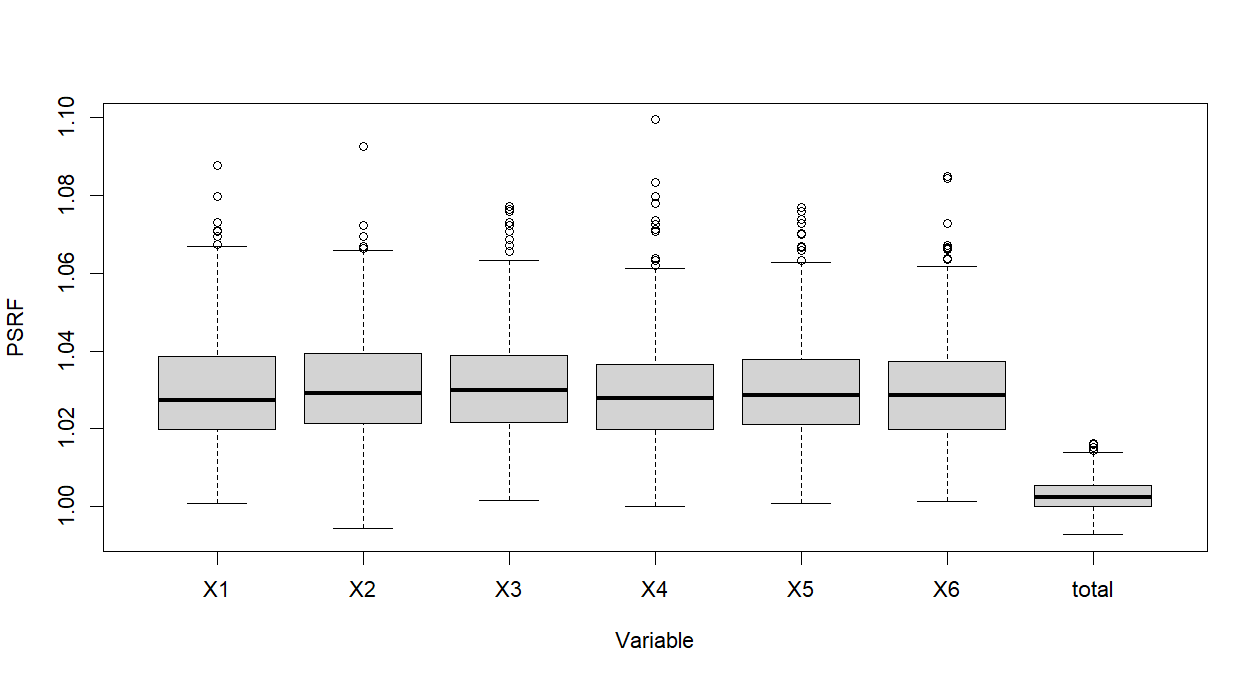


**Figure S3:** PSR statistics of items X1 – X6 and total score at *t* = 5 and the last iteration across 50 chains in 500 replicates.

**Figure S4** shows the bias, SD and empirical power for 5 methods across replicates 100 – 1000. All evaluation metrics stabilized after 500 replicates, indicating good convergence at this threshold.


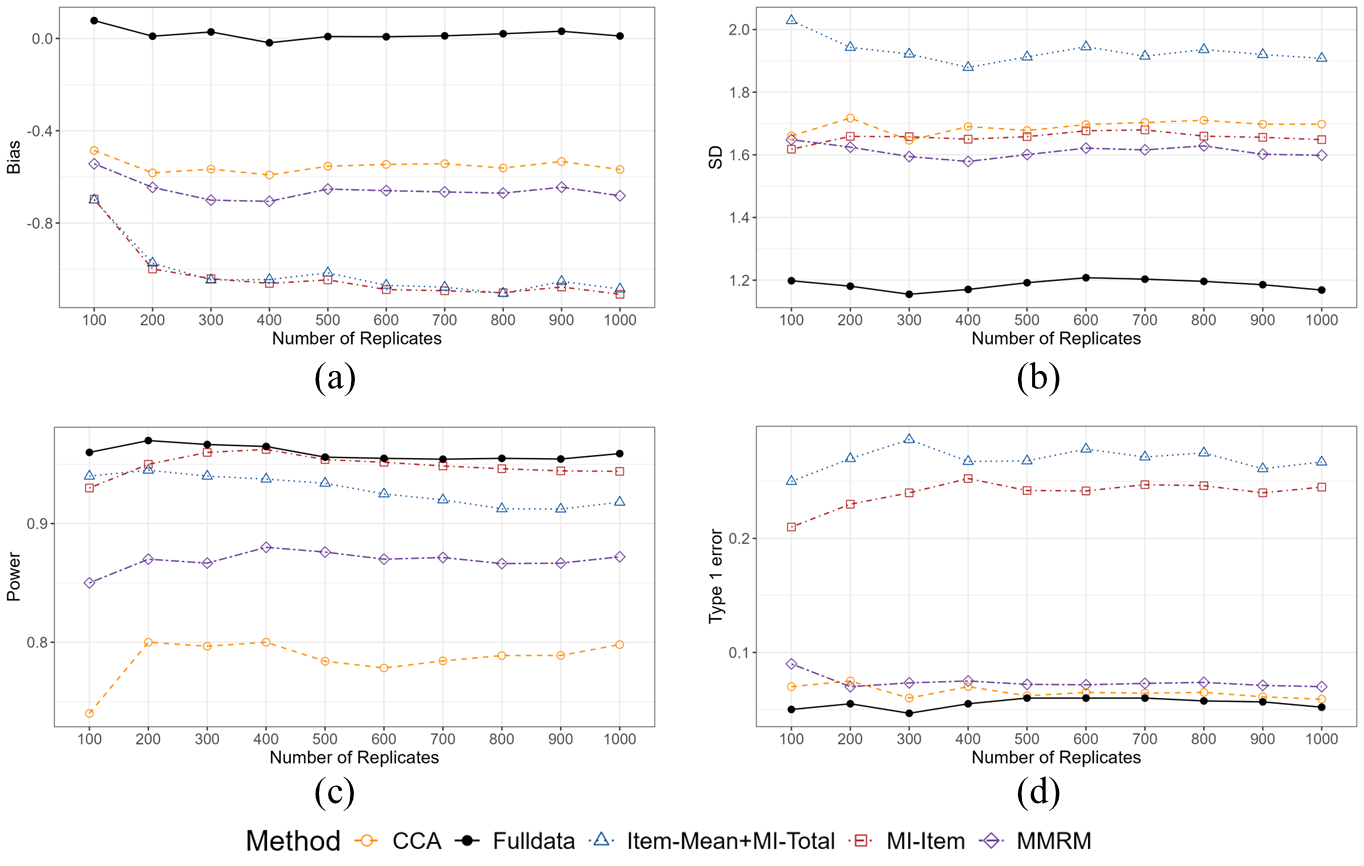


**Figure S4:** Bias, SD and power across replicates 100 – 1000：(a) Bias; (b) SD; (c) Power; (d) Type 1 error.

## Simulation Results


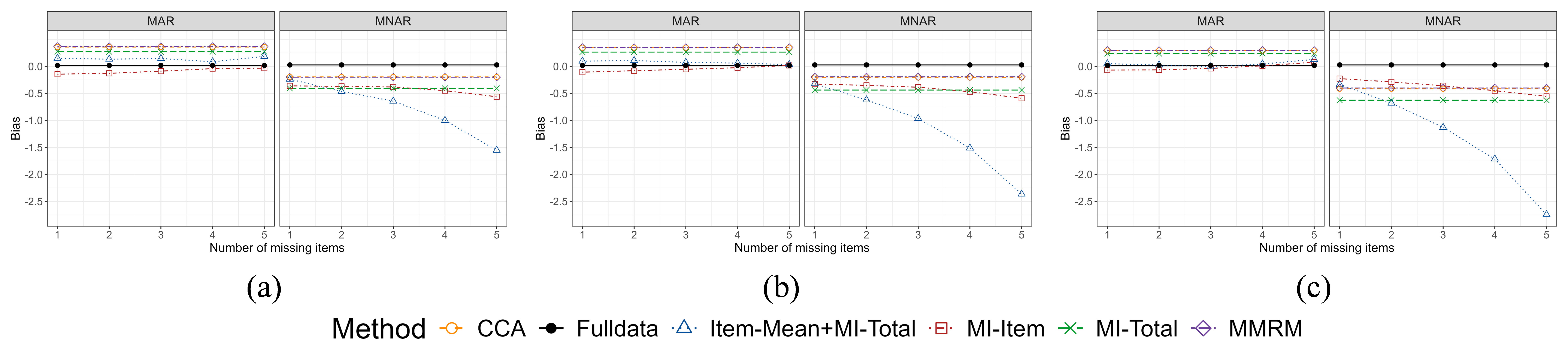


**Figure S5:** Bias under simple questionnaire structure under fixed numbers of missing items with varying item and visit missing rates: (a) missing item rate = 0.1, missing visit rate = 0.3, (b) missing item rate = 0.2, missing visit rate = 0.2 and (c) missing item rate = 0.3, missing visit rate = 0.1.


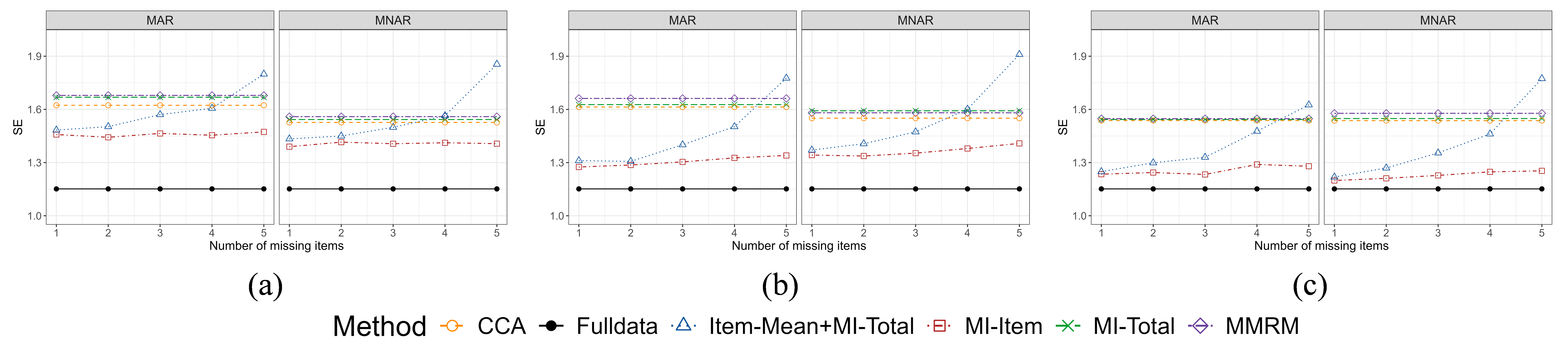


**Figure S6:** SE under simple questionnaire structure under fixed numbers of missing items with varying item and visit missing rates: (a) missing item rate = 0.1, missing visit rate = 0.3, (b) missing item rate = 0.2, missing visit rate = 0.2 and (c) missing item rate = 0.3, missing visit rate = 0.1.


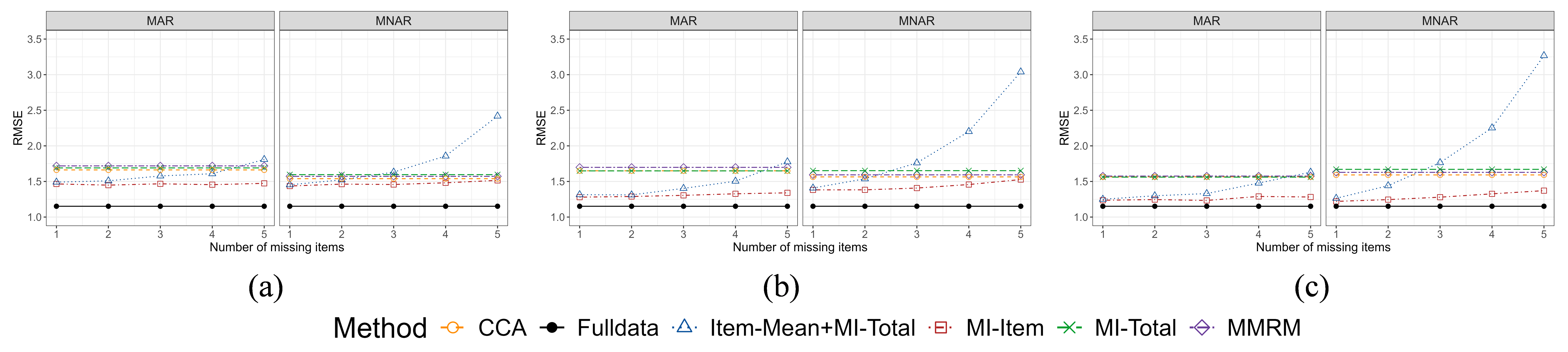


**Figure S7:** RMSE under simple questionnaire structure under fixed numbers of missing items with varying item and visit missing rates: (a) missing item rate = 0.1, missing visit rate = 0.3, (b) missing item rate = 0.2, missing visit rate = 0.2 and (c) missing item rate = 0.3, missing visit rate = 0.1.


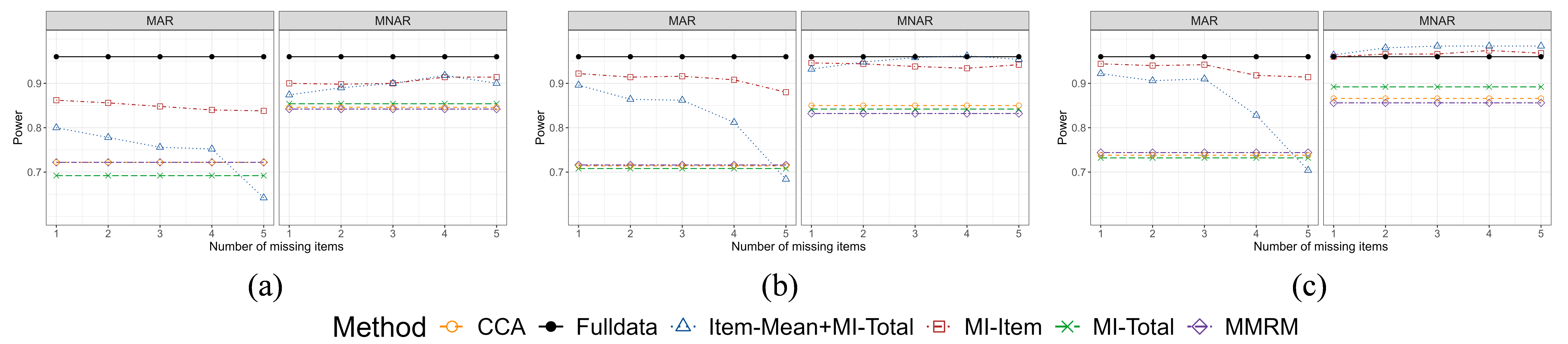


**Figure S8:** Power under simple questionnaire structure under fixed numbers of missing items with varying item and visit missing rates: (a) missing item rate = 0.1, missing visit rate = 0.3, (b) missing item rate = 0.2, missing visit rate = 0.2 and (c) missing item rate = 0.3, missing visit rate = 0.1.


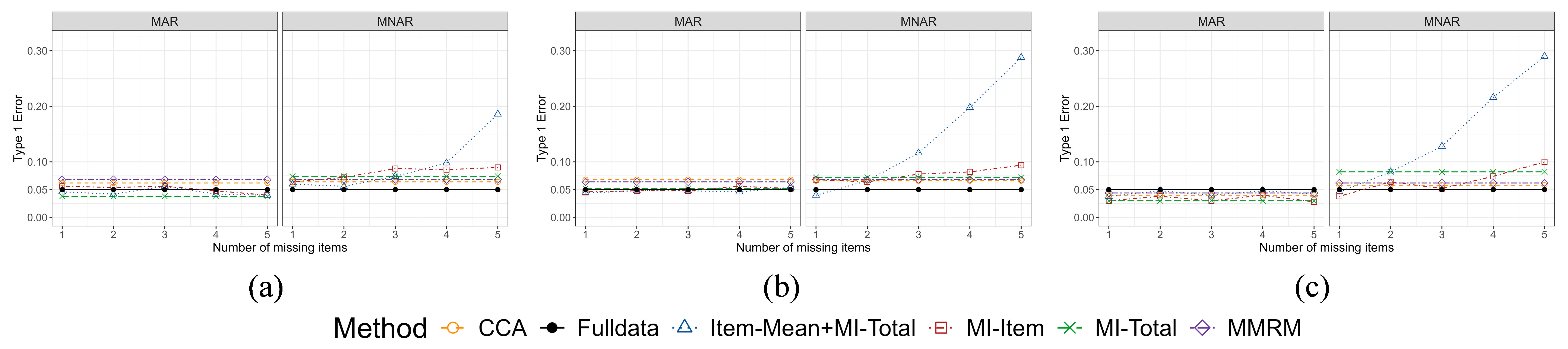


**Figure S9:** Type 1 error under simple questionnaire structure under fixed numbers of missing items with varying item and visit missing rates: (a) missing item rate = 0.1, missing visit rate = 0.3, (b) missing item rate = 0.2, missing visit rate = 0.2 and (c) missing item rate = 0.3, missing visit rate = 0.1.

#### **Table S4** Results under complex questionnaire structure, unfixed number of missing items: Comparison of varying missing item rates and missing visit rates, n=200, MNAR

| **Missing Visit Rate** | **Method** | **Missing Item Rate=0.1** | | | **Missing Item Rate=0.2** | | |
| --- | --- | --- | --- | --- | --- | --- | --- |
|  |  | **RMSE (%)** | **Power** | **Type 1 Error** | **RMSE (%)** | **Power** | **Type 1 Error** |
| 0.2 | HUM+MMRM | 1.626 (122%) | 0.890 | 0.048 | 1.625 (122%) | 0.900 | 0.044 |
|  | MI-Item | **1.573 (118%)** | **0.936** | 0.060 | **1.515 (114%)** | **0.954** | 0.068 |
|  | HUM+MI-Total | 1.679 (126%) | 0.898 | **0.032** | 1.682 (126%) | 0.896 | **0.040** |
|  | HUM+MI-Domain | 1.585 (119%) | 0.934 | 0.060 | 1.549 (116%) | 0.950 | 0.050 |
|  | CCA | 1.646 (123%) | 0.874 | 0.046 | 1.685 (126%) | 0.842 | 0.048 |
|  | Full data | 1.333 (100%) | 0.958 | 0.050 | 1.333 (100%) | 0.958 | 0.050 |
| 0.3 | HUM+MMRM | 1.673 (126%) | 0.848 | 0.062 | 1.750 (131%) | 0.844 | **0.066** |
|  | MI-Item | **1.617 (121%)** | **0.930** | 0.072 | **1.661 (125%)** | 0.922 | 0.074 |
|  | HUM+MI-Total | 1.734 (130%) | 0.864 | **0.054** | 1.816 (136%) | 0.870 | 0.074 |
|  | HUM+MI-Domain | 1.618 (121%) | 0.928 | 0.084 | 1.672 (125%) | **0.924** | 0.084 |
|  | CCA | 1.691 (127%) | 0.822 | 0.048 | 1.863 (140%) | 0.768 | **0.066** |
|  | Full data | 1.333 (100%) | 0.958 | 0.050 | 1.333 (100%) | 0.958 | 0.050 |

#### **Table S5** Results under complex questionnaire structure and unfixed number of missing items: Comparison of varying sample sizes, missing visit rate = 0.3, missing item rate = 0.2, MNAR

| ***n*** | **Method** | **RMSE (%)** | **Power** | **Type 1 Error** |
| --- | --- | --- | --- | --- |
| 100 | HUM+MMRM | 2.526 (131%) | 0.536 | 0.070 |
|  | MI-Item | **2.123 (110%)** | 0.640 | 0.054 |
|  | HUM+MI-Total | 2.402 (124%) | 0.528 | 0.044 |
|  | HUM+MI-Domain | 2.231 (116%) | **0.718** | 0.090 |
|  | CCA | 2.691 (139%) | 0.450 | **0.036** |
|  | Full data | 1.930 (100%) | 0.704 | 0.050 |
| 200 | HUM+MMRM | 1.750 (131%) | 0.844 | 0.066 |
|  | MI-Item | **1.660 (125%)** | 0.922 | 0.074 |
|  | HUM+MI-Total | 1.816 (136%) | 0.870 | 0.074 |
|  | HUM+MI-Domain | 1.672 (125%) | **0.924** | 0.084 |
|  | CCA | 1.863 (140%) | 0.768 | **0.066** |
|  | Full data | 1.333 (100%) | 0.958 | 0.050 |
| 400 | HUM+MMRM | 1.186 (130%) | 0.990 | **0.046** |
|  | MI-Item | **1.168 (128%)** | **1.000** | 0.096 |
|  | HUM+MI-Total | 1.796 (197%) | 0.838 | 0.056 |
|  | HUM+MI-Domain | 1.712 (188%) | 0.942 | 0.148 |
|  | CCA | 1.261 (138%) | 0.974 | 0.056 |
|  | Full data | 0.911 (100%) | 1.000 | 0.050 |

# S3. Supportive Information of the Analysis on Trial Data

The FACT-P questionnaire consists of 39 items in five domains (physical well-being [PWB), social/family well-being [SWB], emotional well-being [EWB], functional well-being [FWB], and prostate cancer subscale [PCS]), and three higher level summary scores (functional assessment if cancer therapy – prostate [FACT-P], functional assessment of cancer therapy [FACT-G] and trial outcome index [TOI]) (See Figure S5).


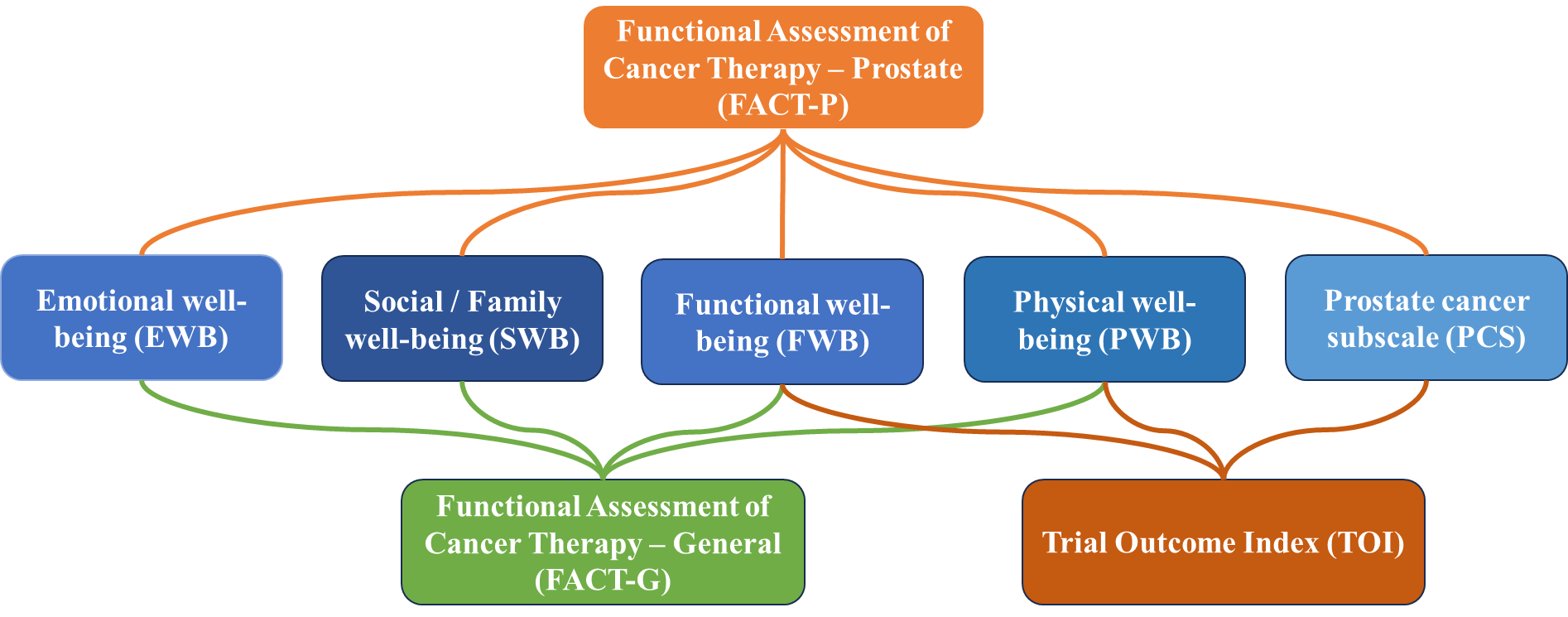


#### **Figure S10** Structure of the FACT-P questionnaire

#### **Table S6** Baseline characteristics (AD-1)

| **Baseline Variable** | **Treatment Group (n=269)** | **Control Group (n = 270)** | ***P*-value** |
| --- | --- | --- | --- |
| Age (mean ± SD) | 67.4 ± 8.92 | 67.1 ± 8.71 | 0.21 |
| ECOG |  |  | 0.38 |
| 0 | 218 | 212 |  |
| 1 | 51 | 58 |  |
| Bone metastasis |  |  | 0.48 |
| Yes | 126 | 124 |  |
| No | 143 | 146 |  |
| FACT-P total score (mean ± SD) | 123 ± 15.4 | 123 ± 17.2 | 0.84 |

#### **Figure S11** Boxplot of FACT-P total score over time (AD-1)


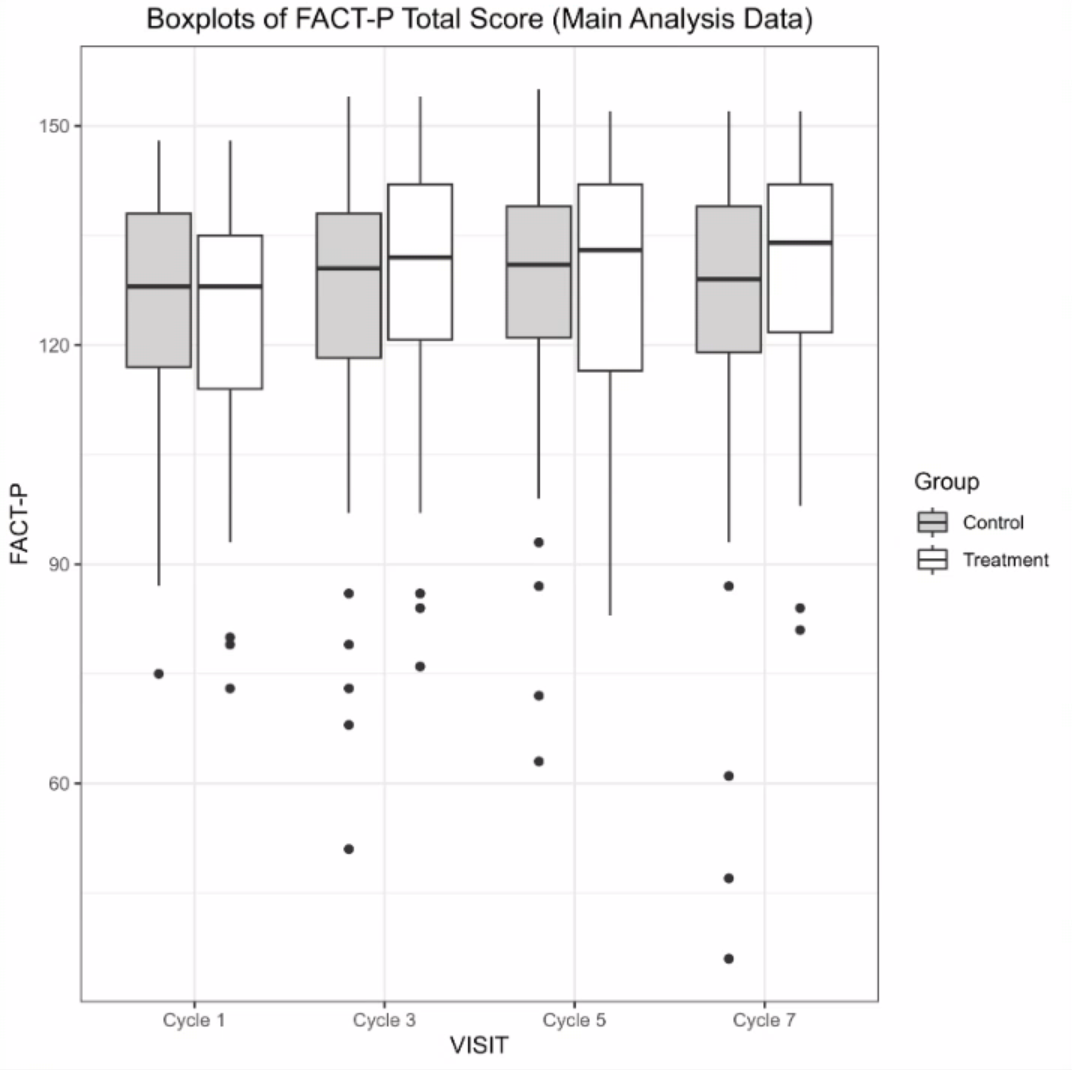


#### **Figure S12** Boxplot of CFB in FACT-P total score over time (AD-1)


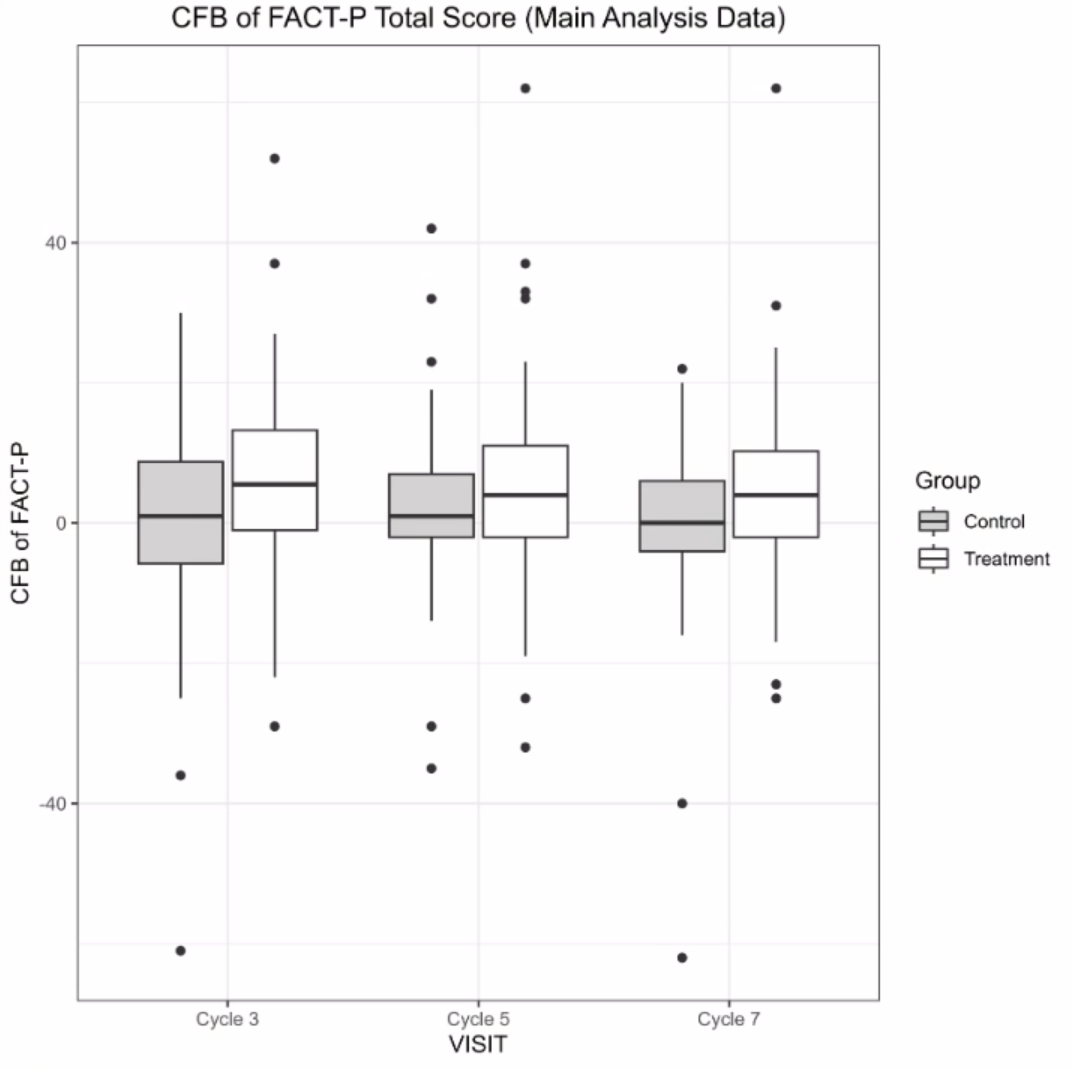


#### **Figure S13** Boxplot of FACT-P total score (AD-2)


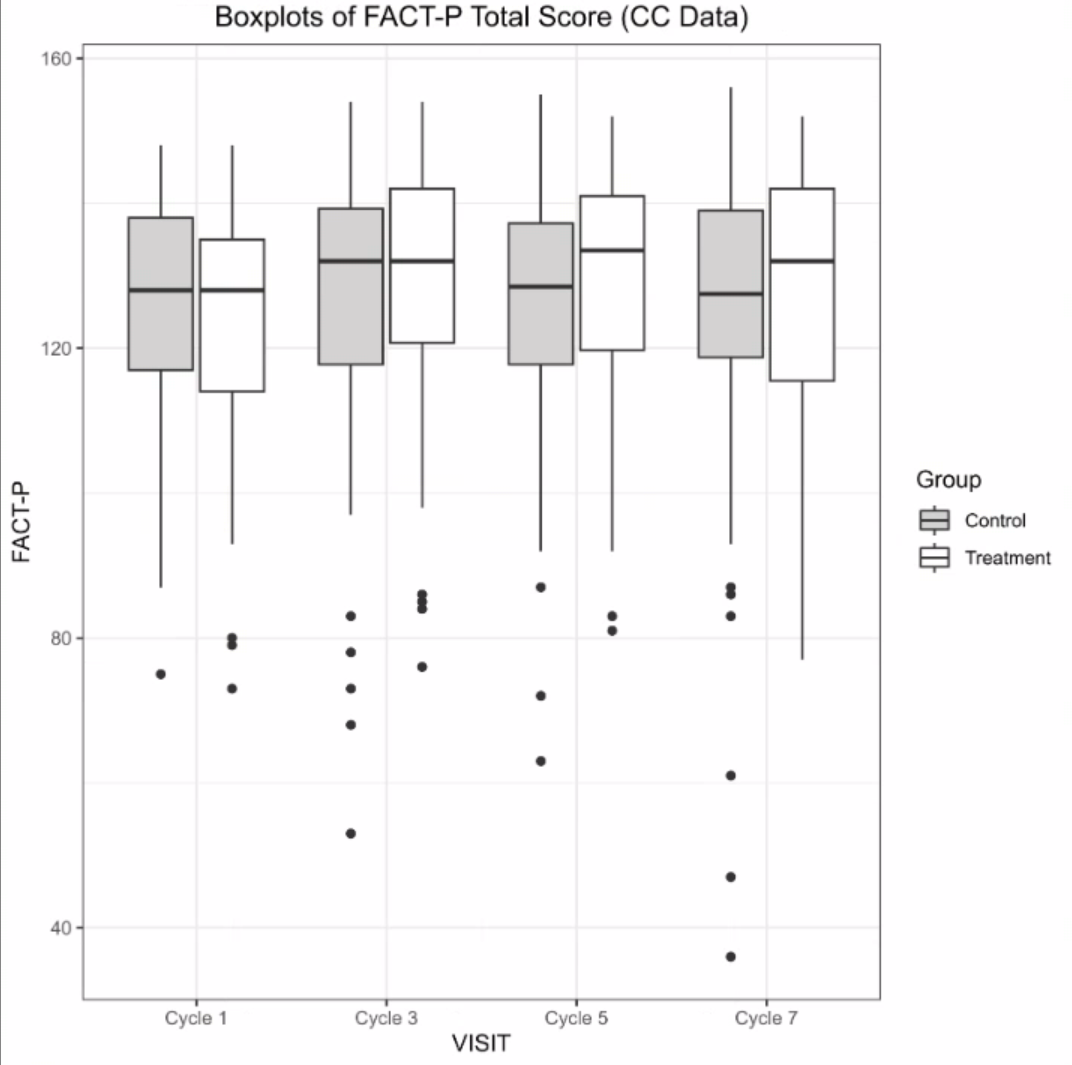


#### **Figure S14** Boxplot of CFB in FACT-P total score over time (AD-2)


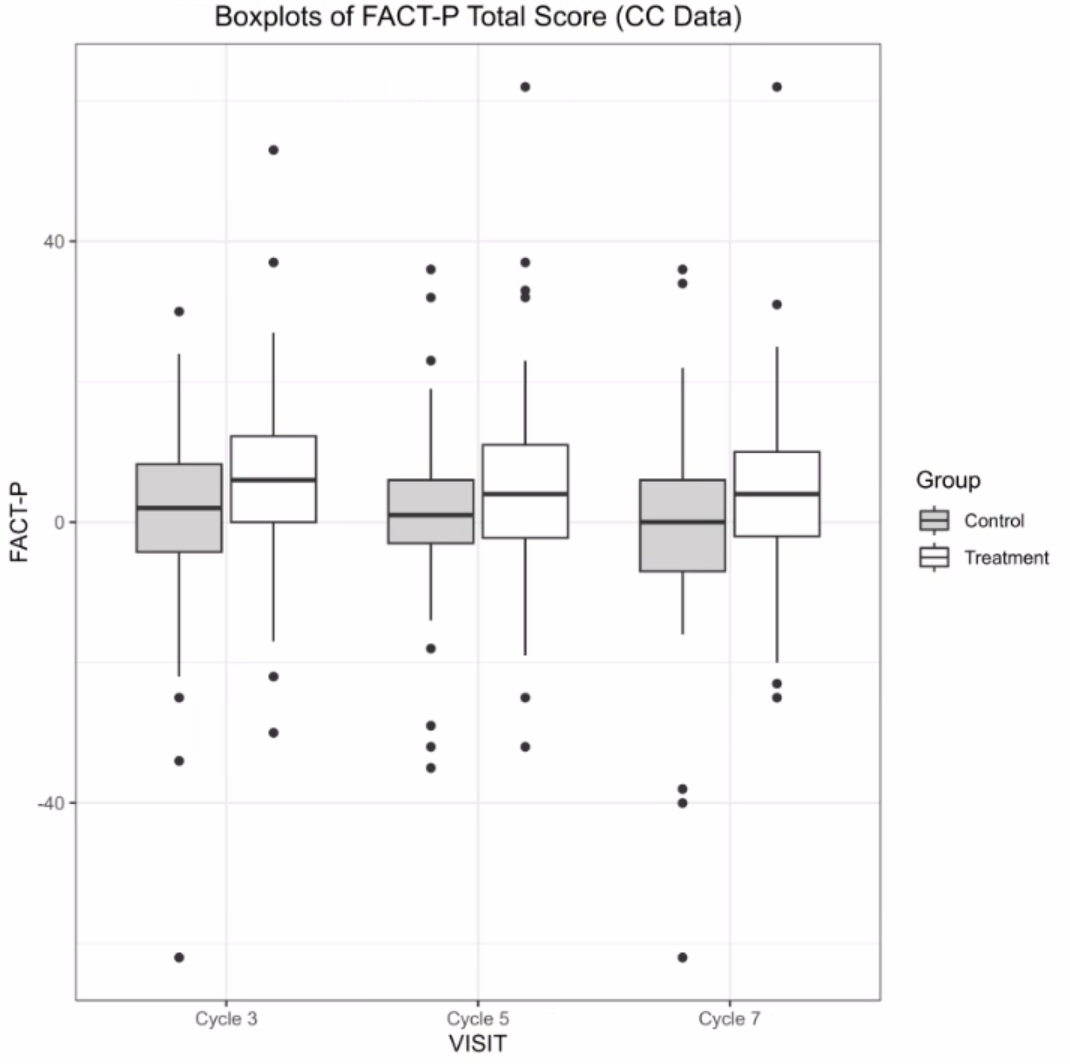


Evaluation criteria in AD-2 Analysis: RMSIE and RMSIE%

Let $y_{i}$, $i=1,2,\ldots,n$ be the true score value and $\hat{y}_{i}^{(k)}$, $k=1,2,\ldots,m$, be the $k$th imputed value, where $n_{0}$ is the number of subjects with imputed values and $m$ is the number of imputed datasets ($m=5$ in our simulation).

To evaluate the difference of imputed values and the true values, RMSIE was defined as follows:

$$RMSIE=\sqrt{\frac{1}{n_{0}}\sum_{i=1}^{n_{0}} \left( \frac{1}{m}\sum_{k=1}^{m} \hat{y}_{i}^{\left( k \right)}-y_{i} \right)^{2}},$$

and RMSIE% was fined as follows,

$$RMSIE\%=\sqrt{\frac{1}{n_{0}}\sum_{i=1}^{n_{0}} \left( \frac{\frac{1}{m}\sum_{k=1}^{m} \hat{y}_{i}^{\left( k \right)}-y_{i}}{y_{i}} \right)^{2}}$$

RMSIE% was used for the comparison between summary scores and domain scores in case they are in different scales.

1. Barnard, J. and D.B. Rubin, *Small-Sample Degrees of Freedom with Multiple Imputation.* Biometrika, 1999. **86**(4): p. 948-955.
